# Acoustic microbubble propulsion, train-like assembly and cargo transport

**DOI:** 10.1038/s41467-023-40387-7

**Published:** 2023-08-05

**Authors:** Jakub Janiak, Yuyang Li, Yann Ferry, Alexander A. Doinikov, Daniel Ahmed

**Affiliations:** Acoustic Robotics Systems Lab (ARSL), Institute of Robotics and Intelligent Systems, ETH Zurich, CH-8803 Rüschlikon, Switzerland

**Keywords:** Acoustics, Mechanical engineering

## Abstract

Achieving controlled mobility of microparticles in viscous fluids can become pivotal in biologics, biotechniques, and biomedical applications. The self-assembly, trapping, and transport of microparticles are being explored in active matter, micro and nanorobotics, and microfluidics; however, little work has been done in acoustics, particularly in active matter and robotics. This study reports the discovery and characterization of microbubble behaviors in a viscous gel that is confined to a slight opening between glass boundaries in an acoustic field. Where incident waves encounter a narrow slit, acoustic pressure is amplified, causing the microbubbles to nucleate and cavitate within it. Intermittent activation transforms microbubbles from spherical to ellipsoidal, allowing them to be trapped within the interstice. Continuous activation propels ellipsoidal microbubbles through shape and volume modes that is developed at their surfaces. Ensembles of microbubbles self-assemble into a train-like arrangement, which in turn capture, transport, and release microparticles.

## Abstract (web summary)

Experiments show how confined microbubbles in a viscous gel can be controllably moved by means of an acoustic field. Ensembles of such microbubbles can be made to self-assemble into a train-like arrangement, which can trap, transport and release microparticles.

## Introduction

Achieving controlled mobility of microparticles and microbubbles in viscous fluids and gel-like media can create exciting new opportunities in the natural and life sciences and open up novel biotechniques and biomedical applications. However, while manipulation of microparticles in viscous gel is both important and challenging^1–11^, little work has been done on this subject. Typically, microparticle mobility has been achieved, generally by (1) applying external-field gradients and (2) instituting nonreciprocal motion, such as a spiraling motion, within a designed microstructure. The first demonstrated manipulation of dielectric microparticles, using optical tweezers, operated on a tightly-focused gradient of light^7,8^. Later, magnetic^9^, acoustic^10–20^, and electric^21^ field gradients were adopted. Acoustic-^22–32^, electric-^33,34^, magnetic-^35–44^, and light-^45^ based approaches can also initiate propulsion by exploiting nonreciprocity within a microstructure or any appendages anchored to it. However, to date, most manipulation and propulsion of microparticles and microarchitectures has been executed in a simple viscous fluid, i.e., water. Although nature’s microswimmers such as bacteria^46^, spirochetes^47^, and spermatozoa^48^ can navigate effectively in complex fluids and gel-like media, their artificial counterparts find viscous fluids extremely challenging. Only a few synthetic microswimmers have achieved navigation in viscous fluids^49,50^, such as a magnetic “micro-scallop” that is propelled through the back and forth, i.e., reciprocal, motion of its appendages^51^. Other magnetic designs have been studied for navigating through bodily fluids^52,53^ and the vitreous humor of the eye^54^. In addition, an acoustic vortex beam was recently developed to trap and manipulate microbubbles inside agarose gels^55^. Another important feature of microrobots is their ability to trap and transport microparticles; however, till date, most artificial swimmers demonstrate trapping in a water-like medium.

Herein we report the discovery of various microbubble behaviors in an acoustic field when confined to shallow openings between two glass boundaries in a shear-thinning gel. We observed microbubble nucleation due to intensification of incident acoustic waves at the narrow slit; theoretical development of the pressure field across the glass boundaries supported this acoustic amplification. When the acoustic field was turned off, the microbubbles moved to the sides. Peculiarly, when dormant microbubbles located outside the opening were exposed to ultrasound, they squeezed through the shallow slit, transforming shape from spherical to discoidal just milliseconds prior, and consequently became trapped. Both single and multiple microbubbles were observed to execute controlled propulsion upon activation, the driving mechanism of which we believe stems from superposition of volume and high-amplitude surface modes developed at the microbubble skin. As individual microbubbles approached each other, they self-assembled into a train and traveled in unison at uniform velocity. Surprisingly, when we injected solid microparticles into the surrounding environment, they became trapped between members of the bubble microtrain. Finally, after the train arrived at a destination, the transducer was deactivated and the trapped microparticles released. Our system thus mimics a cargo train at microscale. We envision that acoustically-activated microbubbles can be a implemented in the position manipulation of microparticles in viscous fluids. The developed platform has a number of prospective uses, such as the controlled manipulation, enrichment, and separation of microparticles in extremely viscous fluids for microfluidic applications and also for applications in biologics and life sciences, for example the investigation of chemotaxis at single-cell resolution in a gel-like medium; extraction and enrichment of cells and exosomes from viscous bodily fluids for lung cancer biomarkers^1,3,6,56–58^; targeted inoculation of cells in gel-mimetic extracellular matrices^59,60^, among others.

## Results

### Experimental setup

**Fig. 1:**
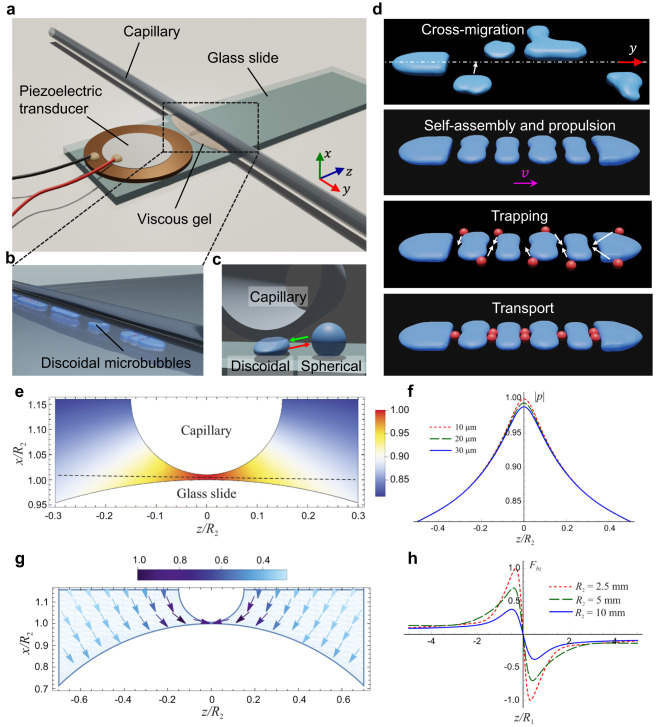
Experimental setup and theoretical model. **a** Schematic of the experimental setup. A piezo transducer is bonded on a glass slide and, when acoustically activated, generates vibration on the slide. A shear-thinning gel is applied 5 to 15 mm from the transducer. Finally, a glass capillary having outer and inner diameters of 1.5 and 1.3 mm, respectively, is positioned on top of the gel, crossing the slide. **b** We investigated microbubble behaviors in the shallow region sandwiched between the glass slide and the capillary. **c** Microbubbles transformed between spherical and discoidal shapes when the acoustic field was turned on (green arrow) or off (red arrow). **d** An illustration of the various behaviors observed: self-assembly, propulsion, trapping, and transport in an acoustically intensified narrow slit. **e** Theoretical calculation of the acoustic field generated between two closely-spaced cylinders surrounded by a liquid. The radial vibration of the bigger cylinder generates an acoustic field, while the smaller cylinder scatters that field. Distribution of the normalized acoustic pressure magnitude in a plane transverse to the cylinders. The radius of the smaller cylinder is ${R}_{1}=0.75$ mm, the radius of the larger cylinder ${R}_{2}=5$ mm, the vibration frequency $f=23$ kHz, the distance between the cylinders’ surfaces (gap width) 50 μm (this value was chosen in order to make the gap between the cylinders visible in the figure), the liquid density ${\rho }_{0}=1086$ kg/m^3^, and the speed of sound in the liquid $c=1450$ m/s. **f** Variation of the normalized acoustic pressure magnitude along the dashed line depicted in (**e**) at three values of the gap width: 10, 20, and 30 μm. **g** Normalized force field experienced by a bubble with radius ${R}_{0}=25$ μm. The distance between the surfaces of the cylinders is 20 μm. **h** The normalized force component${F}_{bz}$, given by Eq. (4), at three values of the radius of the bigger cylinder *R*_2_ for a bubble located on the dashed line depicted in (**e**).

Our experimental design incorporates a piezo disc transducer mounted on a glass slide, as shown in the schematic in Fig. 1a. An electronic function generator drives the transducer to produce vibration in the glass slide. We activated the transducer’s thickness mode at excitation frequencies of 22.3−23 kHz and amplitude 20–40 V_PP_. A viscous, shear-thinning gel was applied to the glass slide ~5–15 mm away from the transducer. A glass capillary with a circular cross-section was then placed on top of the gel and pressed down until bubbles began to nucleate and cavitate in the interstice; see Figs. 1b, c and S1. The entire setup was placed on an inverted microscope connected to a highly sensitive, high-speed camera to study the behaviors of microbubbles within the confines of the narrow aperture (see also Fig. 1d and “Materials and methods”).

### Modeling of acoustic pressure across a narrow slit

A theoretical model across the narrow slit has been developed that explains the physical mechanism behind the experimental effects we observed. The model approximates the physical situation under study as follows. It is assumed that there are two closely-spaced cylinders, of which the bigger cylinder, with radius *R*_2_, vibrates radially, generating an acoustic field in the surrounding liquid. Note that the glass slide used in our experiments is approximated as a cylinder whose radius tends to infinity. The smaller cylinder (the capillary), with radius *R*_1_, scatters the acoustic field generated by the bigger cylinder. The acoustic pressure produced by the cylinders is calculated by $p=-{\rho }_{0}\partial \varphi /\partial t$, where *ρ*_0_ is the equilibrium liquid density and *φ* is the potential of the liquid velocity, which obeys the Helmholtz equation^61^${\nabla }^{2}\varphi+{k}^{2}\varphi=0$, where $k=\omega /c$ is the wavenumber, *ω* is the angular frequency, and *c* is the speed of sound in the liquid. The calculation gives:

1 $$p(x,z,t)=i\omega {\rho }_{0}{e}^{-i\omega t}\mathop{\sum }\limits_{n=-\infty }^{\infty }[{a}_{n}^{(1)}{H}_{n}^{(1)}(k{r}_{1}){e}^{in{\theta }_{1}}+{a}_{n}^{(2)}{H}_{n}^{(1)}(k{r}_{2}){e}^{in{\theta }_{2}}],$$

where ${H}_{n}^{(1)}$ is the Hankel function of the first kind, $({r}_{j},{\theta }_{j})$ is the polar coordinates originated at the center of the *j*th cylinder (*j* = 1, 2), and the coefficients ${a}_{n}^{(j)}$ are calculated by boundary conditions at the cylinders’ surfaces (see Supplementary Section 1.1). The developed theoretical model is valid for any separation distances between cylinders, which allows us to calculate the pressure field at narrow slits. The viscous boundary layer ($\delta=\surd (2\nu /\omega )$, where *ν* is the kinematic viscosity of the liquid, which is of the order of 6 μm in our case, so it does not affect the scattered acoustic field produced by the cylinders in the bulk of the liquid.

Figure 1e shows the distribution of |*p*| in a plane transverse to the cylinders, suggesting an amplification of the acoustic field at the interstice. The plotted values are normalized to the maximal pressure value, which occurs at *z* = 0, i.e., the midpoint between the cylinders (Fig. 1f). Calculations revealed that, qualitatively, the behavior of the acoustic pressure remains the same regardless of the cylinders’ radii and the distance between them. We estimated the pressure at the slit to be about 100 kPa based on measurements taken with a laser doppler vibrometer (see also Supplementary Figs. S2 and S3).

### Microbubble formation and migration

We first studied the nucleation and cross-migration of microbubbles in a viscous gel through a narrow gap between a capillary and the glass slide. Based on our theoretical model, when an acoustic wave was triggered at a frequency of 23 kHz and voltage of 20 V_PP_, a pressure field formed and was intensified in the space between, which initiated the microbubble nucleation process. Nucleation of microbubbles occurs at the gas-liquid interface at the slit, which initiates at the edge of the glass slide when an acoustic field is present. Figure S4 shows the distribution of gas nuclei in the slit region when the piezo electric transducer is activated (see also Supplementary Video 1). Our experiments have shown that microbubbles of different sizes are produced across the slit, with the smallest arising at the center and larger ones as we move radially across the slit, see also Fig. S5 and Supplementary Video 2. Conversely, when continuously exposed to acoustic radiation for seconds, the microbubbles grow rapidly until reaching the critical size at which it comes into contact with the bounding surfaces of the capillary and the glass slide. Figure S6 illustrates the size and shape of the microbubble versus driving power. The microbubble then conforms to those surfaces, causing it to become discoidal in shape and eventually stabilize into a bubble train. The unbalanced acoustic pressure distribution within the gap, i.e., the high-pressure gradient along the +*z* and −*z*-axis, further squeezes the microbubble along the *z*-axis, thus giving rise to an ellipsoidal microbubble with its major axis oriented in the *y*-direction. When the acoustic field is turned off, the ellipsoidal microbubbles revert to spherical shape and are squeezed out of the interstice, usually within seconds, migrating to either side of the capillary in the direction of the *z*-axis (see also Supplementary Video 3). It is quite intriguing that when we subsequently re-activated the acoustic field, the microbubbles squeezed back into the slit and reverted to a discoidal shape in milliseconds.

Knowing the acoustic field produced by the cylinders makes it possible to calculate the acoustic radiation force^62^ that acts on a gas bubble situated beside them (Supplementary Section 1.2). The force is defined by the following equation:

2 $${{{{{{\boldsymbol{F}}}}}}}_{b}=-\frac{4}{3}\pi \langle {R}^{3}{{{{{\boldsymbol{\nabla }}}}}}p\rangle,$$

where $\langle \,\rangle$ means the time average, *R* is the instantaneous bubble radius, which is calculated by the Rayleigh–Plesset equation^63,64^, and *p* is the acoustic pressure calculated by Eq. (1). The calculation shows that the radiation force has *x* and *z* components, which are given by:

3 $${F}_{bx}=2\pi {\rho }_{0}k{R}_{0}{{{{\mathrm{Re}}}}}\left\{\frac{{G}_{x}(x,z)}{{\omega }_{0}^{2}/{\omega }^{2}-1+i\delta }\right\},$$

4 $${F}_{bz}=2\pi {\rho }_{0}k{R}_{0}{{{{\mathrm{Re}}}}}\left\{\frac{{G}_{z}(x,z)}{{\omega }_{0}^{2}/{\omega }^{2}-1+i\delta }\right\},$$

and lies in the plane transverse to the cylinders. Here, *R*_0_ is the bubble’s equilibrium radius, Re means “the real part of”, *ω*_0_ is the bubble’s resonance frequency, *δ* is the damping constant of the bubble’s oscillation, (*x*, *z*) are the coordinates of the bubble’s center, and the functions *G*_*x*_(*x*, *z*) and *G*_*z*_(*x*, *z*) which only depend on the acoustic field at the point (*x*, *z*), are defined by Supplementary Eqs. (1.40) and (1.41).

Figure 1g, h illustrates predictions of Eqs. (3) and (4), which were calculated with values corresponding to our experimental parameters: ${R}_{1}=0.75$ mm, the distance between the cylinders’ surfaces 20 μm, the vibration frequency $f=23$ kHz, ${\rho }_{0}=1086$ kg/m^3^, $c=1450$ m/s, and the gas inside the bubble is air. Figure 1g specifically shows the force field experienced by a bubble with radius ${R}_{0}=25$ μm (${f}_{0}={\omega }_{0}/2\pi=128.7$ kHz, where *f*_*o*_ is the resonance frequency of the microbubble, Eq. 1.31 in Supplementary Information) in the case where ${R}_{2}=5$ mm, while Fig. 1h depicts the value of the force component *F*_*bz*_ for various values of *R*_2_ assuming that the bubble is sited on the dashed line depicted in Fig. 1e. The force is normalized to the maximal value. It is readily seen that at $z < 0$, ${F}_{bz} > 0$, while at $z > 0$, ${F}_{bz} < 0$. This means that the radiation force causes the bubble to move to the plane $z=0$, where ${F}_{bz}=0$; that is, it moves to the space between the cylinders and settles there. So long as the acoustic field is on, the position of the bubble at $z=0$ is stable; if the bubble becomes displaced by random perturbations, the radiation force returns it to this position. It should be emphasized that Fig. 1e–h are strictly concerned with small bubbles, i.e., those with ${\omega }_{0}/\omega > 1$, since our experiments employed such bubbles. In the case depicted, ${\omega }_{0}/\omega=5.6$.

### Discoidal microbubble propulsion

In 1932, Gaines^65^ first noticed a peculiar phenomenon: the erratic behavior of gas-filled bubbles in a liquid after being irradiated with intense ultrasound^65,66^. This randomly directed, incredibly fast-moving behavior, dubbed the “dancing bubble” phenomenon, is often difficult to observe, and despite extensive theoretical development, its controlled manipulation has remained difficult to achieve.

**Fig. 2:**
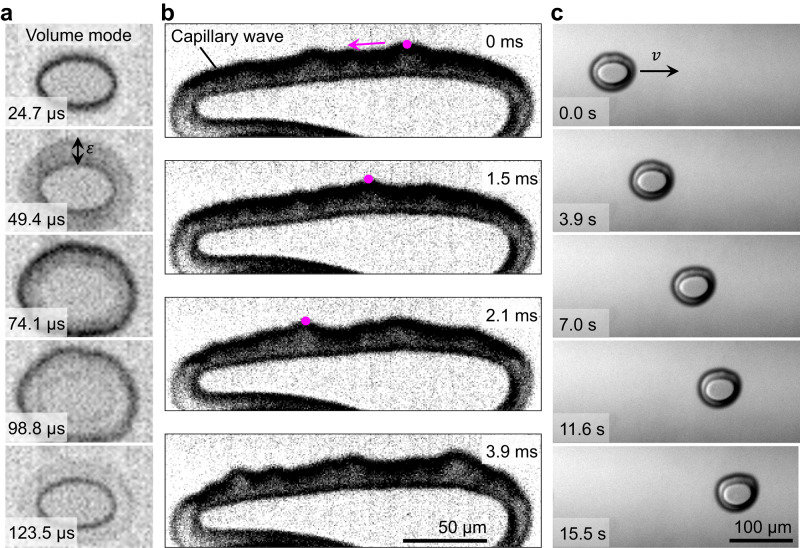
Oscillation modes and propulsion of ellipsoidal microbubbles in ultrasound. **a** Image sequences demonstrating the volume mode oscillation of an ellipsoid microbubble trapped inside the narrow slit. A large oscillation amplitude (*ε*) of ~10 μm was observed under the bright field microscope. **b** A sequence of high-speed images taken at 40,000 frames per second illustrating the shape modes developed on the surface of an elongated microbubble. The capillary wave travels to the left, as indicated by the magenta dot on the crest of the wave. **c** Image sequences illustrating the translational motion of a single ellipsoidal microbubble through hydrogel inside a narrow slit when exposed to ultrasound.

With application of an acoustic field, an ellipsoidal microbubble undergoes a volume mode oscillation with amplitude of ~10 μm that is superimposed with shape modes on the surface of the gas microbubble, as shown in Fig. 2a, b and Supplementary Videos 4 and 5. In the case of our narrowly-separated capillary and slide setup using a viscous shear-thinning fluid, this resulted in single and multiple microbubbles propelling along the gap in the *y*-direction, as depicted in the image sequences of Fig. 2c (see also Supplementary Video 6). The acoustic pressure distribution developed in the interstice, as modeled in Fig. 1f, thus mimics a rail, preventing any sideways deviation of the microbubbles. Initially, we expected that increasing the power applied to the system would cause the bubbles to propel faster; instead, we observed increased power to result in the ellipsoidal microbubbles becoming unstable, behaving erratically or merging with other bubbles, see Supplementary Video 7.

The propulsion of microbubbles in the *y*-direction, i.e., along the gap between the glass slide and the capillary, can be explained as follows. First, the results of Doinikov et al.^67^ suggest that a standing acoustic wave is generated with wave vector direction along *y* in the intervening fluid layer between the glass slide and the capillary, and a velocity node sits beneath the center of the capillary. Then, the dispersion equation derived by Doinikov et al.^67^ predicts that, at 23 kHz and given a 20 μm narrow slit between the glass slide and the capillary, this standing wave should have a wavelength of 5.6 cm. The translational motion of the microbubbles can be explained by an acoustic radiation force exerted on the microbubbles by that standing wave. The classical Bjerknes force^68,69^, which occurs in a weak acoustic field, cannot be responsible for the observed propulsion because that force is directed toward a velocity node if the bubble is driven below resonance ($\omega < {\omega }_{0}$), as in our case^68,69^. In our experiment, microbubbles could propel away from the velocity node both to the right and to the left. Since the capillary acts as a rigid reflector, the pressure magnitude of the acoustic field in the gap between the slide and the capillary can be sufficiently high, which leads to the excitation of shape oscillation modes on the bubble surface^68^. The interaction of these modes gives rise to an additional radiation force on the bubble, which counteracts the classical Bjerknes force and makes the bubble move away from the velocity node, i.e., toward the edges of the capillary^67^. Therefore, the bubble train can move in both directions (Fig. S7).

### Chain-like assembly and propulsion of microbubbles

**Fig. 3:**
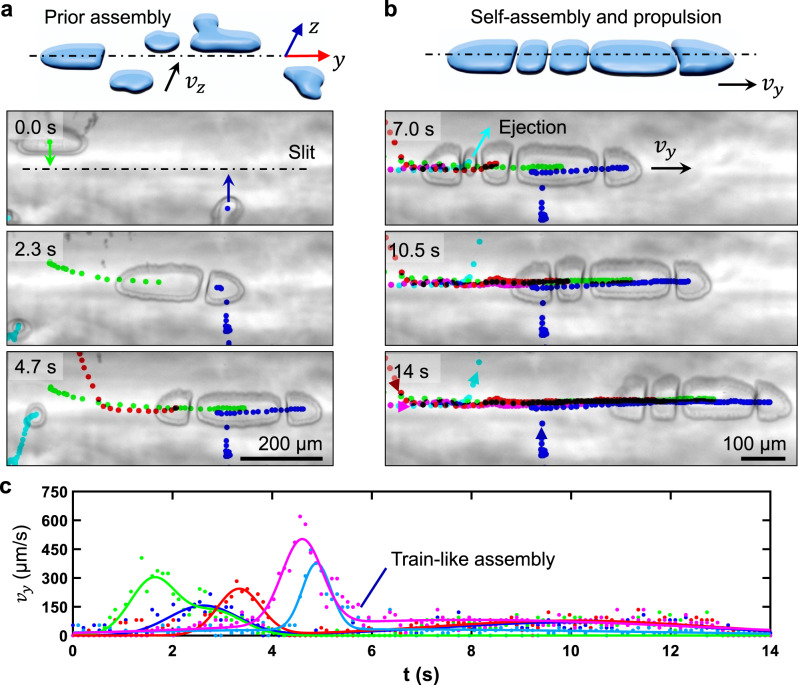
Self-assembly and propulsion of ellipsoidal microbubbles. **a** Schematic and image sequence depicting the microbubbles crawling in the *z*-direction and squeezing into the narrow slit when exposed to ultrasound. These microbubbles then self-assemble into train-like arrangements. The arrows indicate the direction of movement of ellipsoidal microbubbles. **b** Image series demonstrating propulsion of the bubble train, translating left-to-right in the narrow slit. **c** Plot of the translational velocities (*v*_*y*_) of microbubbles moving along the narrow slit in the *y*-direction.

When exposed to ultrasound, microbubbles crawled in the *z*-direction and squeezed into the narrow slit, becoming ellipsoidal. The pressure gradient developed across the aperture caused them to accelerate, increasing cross-migration velocity (*v*_*z*_). The microbubbles subsequently propel themselves along the capillary, translating at different velocities (*v*_*y*_), but also tend to be drawn together by the secondary Bjerknes force. Specifically, when one microbubble approaches an adjacent microbubble, its velocity (*v*_*y*_) abruptly increases until a limit of proximity is reached and they are required to settle into an equilibrium velocity. Ultimately, this leads the microbubbles to become arranged into a quasi-microtrain, which could propel over long distances (~3.5 mm) at a remarkable speed of up to 0.6 mm/s; see also Fig. S8 and Supplementary Video 8. The overall length of the structure determined by the number of microbubbles, as illustrated in Fig. 3a, b and Supplementary Video 9.

We then investigated the number of microbubbles in a train as a function of excitation voltage. Figure S9 demonstrates that the bubble count varies between five and six as the acoustic voltage increases from 20 to 40 V_PP_ at 22.6 kHz. However, no direct correlation with increasing voltage was observed. Next, we investigated the number of microbubbles in a train versus excitation frequency. The microbubbles in the train-like arrangement are produced at certain excitation frequency bands, which coincide with the piezo transducer’s resonance frequencies. Figure S9b illustrates that the bubble count in a train remains between five and six, and is independent of the excitation frequency. We further characterized the interparticle distance *d* between adjacent microbubbles in a train-like assembly for a sweeping voltage of 16.8 to 18 V_PP_ at a fixed excitation frequency of 23.2 kHz. Figure S10 illustrates that with increasing piezotransducer voltage, the interparticle distance between adjacent microbubbles in the train decreases, see also Supplementary Video 10. Among the inner members of the train, a strong repulsive force is manifested, causing a striking gap length of ~10 µm between adjacent microbubbles; they do not coalesce. In addition, the inner microbubbles tended to deform from ellipsoidal into almost rectangular shapes and barely oscillate in the *y*-direction, whereas the leading and trailing microbubbles oscillated freely. Despite the apparent repulsion between adjacent microbubbles, a set of bubbles lock themselves together and travel in unison, as illustrated in Fig. 3c. Occasionally, microbubbles were observed to eject from the train. This usually occurred when an interjacent microbubble was significantly smaller than those on either side, as shown by the microbubble tracked with cyan pigment in Fig. 3b.

### Trapping and transport mechanism

**Fig. 4:**
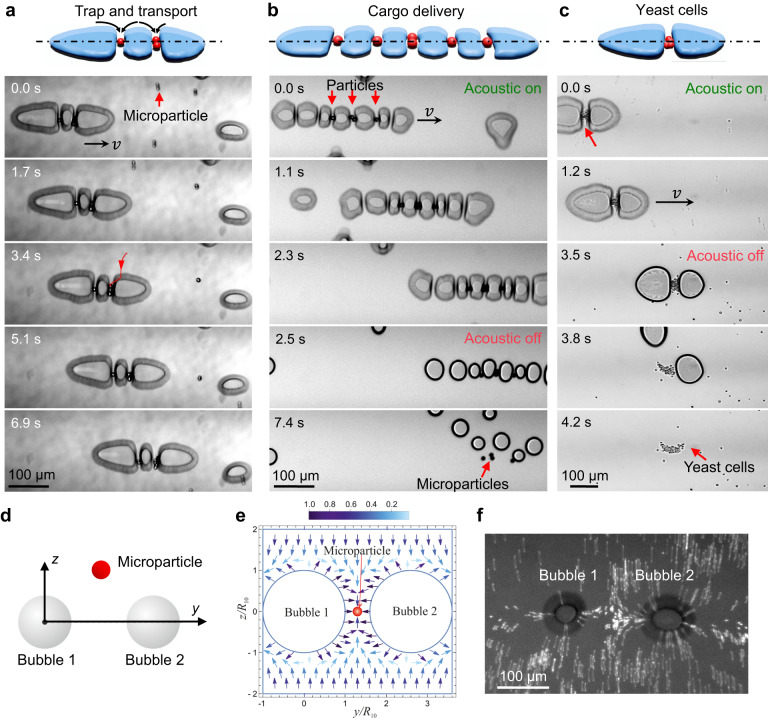
Trapping and transport mechanism. The schematic and image sequences demonstrate (**a**) bubble train propulsion and simultaneous trapping of solid particles at the center point between adjacent microbubbles. **b** The transport and release of microparticles at 2.5 s when the acoustic field is deactivated. Microbubbles emerge from a narrow slit leaving solid particles behind. **c** The trapping, transport, and release of yeast cells, at 3.5 s when the acoustic field is deactivated. **d** A solid microparticle bounded by two oscillating bubbles in an acoustic field. **e** Force field experienced by the microparticle was calculated using the following parameters. The equilibrium bubble radii are ${R}_{10}={R}_{20}=25$ μm. The distance between the bubbles’ surfaces is 15 μm. The bubbles oscillate at 23 kHz. The particle, with radius ${R}_{p}=5$ μm, is made of polystyrene, whose density and sound speed are ${\rho }_{p}=1060$ kg/m^3^ and *c*_*p*_ = 2350 m/s. The sound speed in the liquid is *c* = 1450 m/s. The liquid density is ${\rho }_{0}=1086$ kg/m^3^. **f** Acoustic streamlines created by oscillation of a pair of discoidal microbubbles illustrates the repulsion between them.

Next, we investigated the trapping behavior of the microbubble train in an acoustic field. We mixed 10-µm polystyrene microbeads with a shear-thinning gel, then sandwiched the resulting solution between the glass slide and capillary. Upon applying an acoustic field, a bubble microtrain was produced. The oscillations of the bubbles subjected the microbeads in the surrounding environment to radiation forces, causing them to be attracted toward the bubbles. As the microparticles approached, they came in contact with the top or bottom surfaces of the microbubbles, then slid left or right along the crosswise centreline until reaching the bubble’s equator. The particles then migrated forward or back and became trapped between bubbles, as depicted in Fig. 4a (see also Supplementary Video 11). Moreover, we observed that solid microparticles do not tend to transverse the interstice; rather, they begin to migrate only in the presence of ellipsoidal microbubbles, approaching the bubble with a velocity that scales inversely to distance, as shown in Fig. S11. Minimum microbubble oscillations (*ε* *~* 2 μm) were observed at these regions. No microparticles were observed to be trapped at the top/bottom regions or on the outermost bubbles of the train, which can be attributed to those regions and microbubbles undergoing the largest oscillations, i.e., *ε* ~ 10 μm. This behavior allows for a very reliable trapping mechanism. In addition, the length of the bubble train, i.e., the number of microbubbles, determines the number of microbeads that can be trapped.

Herein we demonstrate the trapping and transport behavior of the bubble train. The image sequence of Fig. 4b illustrates the progression of a bubble train and trapped polystyrene microparticles along a predefined path down the long axis of a capillary. When the bubble train had traveled 345 µm, we turned the acoustic field off. The discoidal-shaped microbubbles ceased oscillating, lacked sufficient energy to remain on the centreline, and so immediately transformed into spherical microbubbles and gradually squeezed out from underneath the capillary. Given the viscous fluid environment, the polystyrene microparticles naturally remained behind at the centreline, and so were separated from the microbubbles (see also Supplementary Video 12). Figure 4c demonstrates the trapping, transport, and release of yeast cells (~4 µm) of the bubble train. We further demonstrated the trapping and transport of polystyrene microparticles (1 and 10 µm) and superparamagnetic particles (2–32 µm) within the microbubbles of the bubble train; see also Fig. S12 and Supplementary Videos 13 and 14.

To explain why solid microparticles are attracted to the space between bubbles, we have calculated the acoustic radiation force that acts on a solid microparticle in the acoustic field generated by the two cylinders and two interacting bubbles placed between the cylinders and undergoing axisymmetric oscillations. The force produced by the cylinders is calculated in Supplementary Section 1.3. To obtain the force produced by the two bubbles, the acoustic field generated by the bubbles is first calculated and then Gor’kov’s formula for the force is used^70^ (Supplementary Section 2). As a result, the following equations for the components of the total radiation force are found (Supplementary Section 3):

5 $${F}_{py}=2\pi {\rho }_{0}{R}_{p}^{3}\left[\frac{{\omega }^{2}{f}_{1}}{3{c}^{2}}{H}_{y}(y,z)-\frac{{f}_{2}}{2}{I}_{y}(\, y,z)\right],$$

6 $${F}_{pz}=2\pi {\rho }_{0}{R}_{p}^{3}\left[\frac{{\omega }^{2}{f}_{1}}{3{c}^{2}}{H}_{z}(y,z)-\frac{{f}_{2}}{2}{I}_{z}(\, y,z)\right],$$

Here ${F}_{py}$ is directed along the centreline of the bubbles (Fig. 4d), ${F}_{pz}$ is perpendicular to the centreline, ${f}_{1}=1-{c}^{2}{\rho }_{0}/({c}_{p}^{2}{\rho }_{p})$, ${f}_{2}=2({\rho }_{p}-{\rho }_{0})/(2{\rho }_{p}+{\rho }_{0})$, ${R}_{p}$ is the particle radius, ${\rho }_{p}$ is the particle density, ${c}_{p}$ is the speed of sound in the particle, $(y,z)$ are the coordinates of the particle center, and the functions ${H}_{y,z}$ and ${I}_{y,z}$, which only depend on the acoustic field at the point $(y,z)$, are defined by Supplementary Eqs. (3.3)–(3.5).

Figure 4e shows the normalized force field of two adjacent microbubbles, which was calculated by Eqs. (5) and (6). The calculation was performed at the following parameters. The equilibrium bubble radii are ${R}_{10}={R}_{20}=25$ μm. The distance between the bubbles’ surfaces is 15 μm. The bubbles undergo radial oscillations with a frequency of 23 kHz. The particle, with radius ${R}_{p}=5$ μm, is assumed to be made of polystyrene, whose density and sound speed are ${\rho }_{p}=1060$ kg/m^3^ and ${c}_{p}=2350$ m/s. The sound speed in the liquid is $c=1450$ m/s and the liquid density is ${\rho }_{0}=1086$ kg/m^3^. The acoustic streamlines created by oscillation of a pair of discoidal microbubbles illustrates the repulsion between them, as illustrated in Fig. 4f (see also Supplementary Video 13). Figure 4e shows that, if the particle gets into the space between the bubbles, the radiation force makes it settle at the midpoint between the bubbles.

It should be noted that in our experiments, we deal with discoidal bubbles. However, there is at present no theory that would allow one to calculate the radiation force produced by such bubbles; the problem is too mathematically complex. Therefore, we have to use theoretical results obtained for spherical bubbles. This approach should be considered an approximation that makes it possible to understand, at least qualitatively, the physical mechanism of the observed effect.

## Discussion

This article demonstrates the concentration of acoustic waves in a narrow slit and the subsequent trapping of microbubbles. We developed a theoretical model of the pressure field at the interstice; the observed amplification of sound waves can be applied in slit-like micro- and nanoarchitectures to capture micro- and nanoparticles. We further demonstrated the controlled propulsion of an ellipsoidal microbubble driven by shape modes superimposed on its volume-mode oscillations. While these initial findings are exciting, further research is needed to determine what controls the direction of propulsion. We have also discovered that upon continuous ultrasound activation, ellipsoidal microbubbles self-assemble into a chain-like arrangement and become propelled in unison. Future work will examine how these ellipsoidal microbubbles and microbubble trains are propelled when confined within a slit set in an arbitrary geometry. Finally, we showed a new and unique mechanism for the trapping and transport of microparticles at central points between microbubbles. We expect our study to launch an alternative strategy for micro- and nanoparticle trapping, particularly in gel-like media. Several biological fluids, including sputum and mucus, are shear-thinning fluids, with shear strengths ranging from 10^−2^ to 10^1^ Pa · s^4^. We have already demonstrated that in these microbubble trains, polystyrene, magnetic particles, and yeast cells can be trapped between adjacent microbubbles in a shear-thinning gel having a viscosity of 0.5 to 100 Pa s. Thus, this concept can be developed to extract and enrich cells from sputum and mucus. More broadly, the spontaneous formation of bubble trains in viscous liquids and their capacity for parallel and uninterrupted capture, transport, and aggregation of bioparticles to designated areas supports the development of an efficient, low-cost, and reagent-free particle-separation method. We identify other applications for such a method: (1) The platform can be used to investigate chemotaxis at a single-cell resolution in a gel-like medium. For example, microparticles that contain chemotactic drugs can be moved to investigate the chemotactic behavior of neutrophils in a viscous gel. (2) The platform could be utilized when seeding cells in a gel that mimics the extracellular matrix, allowing for significant improvements in cellular patterning.

## Methods

### Experimental setup

A piezo transducer is bonded on a glass slide and, when electrically activated, generates vibration waves on the glass slide. We applied viscous KY gel (K-Y Lubricating Jelly Sterile) to the glass slide. The KY gel is an aqueous lubricant containing water and glycerol. The rheological properties of the gel are shown in Fig. S13. The sound speed and the fluid’s density are ~1500 m/s and ~1000 kg/m^3^, respectively^71,72^. Finally, a glass capillary having an outer diameter of 1.5 mm is positioned on top of the gel ~5 mm from the transducer, crossing the slide. The whole setup was positioned on an inverted microscope (ZEISS Axiovert 200 M) and experiment results were captured using a high-sensitivity and high-speed camera. The piezoelectric transducer is connected to a function generator capable of creating waves with a peak-to-peak voltage of 20 V (Tektronix AFG3011C), and is used to generate a square wave with 20 V_PP_ and frequencies between 22.3 and 23 kHz.

### High-speed image acquisition

High-speed images of the bubble oscillations were taken at a framerate of 40,420 fps with an acoustic excitation frequency of 22.3 kHz. Since the bubble oscillation must align with the oscillation of the acoustic field, the period of the oscillation of the bubble should be 45 μs. The 40,420 fps yields a frame length of 25 μs. Consequently, every 1.8 frames of the video are composed of one oscillation cycle of the bubble, and every 9 frames are composed of 5 cycles. These 9 frames were taken from 5 separate cycles at varying stages of the oscillation. By rearranging the frames chronologically according to the cycle progression, we are able to show 1 oscillation cycle of a bubble in 9 frames, as if it were taken at 202,100 frames per second.

### Reporting summary

Further information on research design is available in the Nature Portfolio Reporting Summary linked to this article.

### Supplementary information

Supplementary Video 1

Supplementary Video 2

Supplementary Video 3

Supplementary Video 4

Supplementary Video 5

Supplementary Video 6

Supplementary Video 7

Supplementary Video 8

Supplementary Video 9

Supplementary Video 10

Supplementary Video 11

Supplementary Video 12

Supplementary Video 13

Supplementary Video 14

Description of Supplementary information files

Supplementary information

Reporting Summary

### Source data

Source data

## Data Availability

Data that support the findings of this study are available within the paper, Supplementary Information and Supplementary Data files. Source data are provided with this paper.
