# Supplementary material for: Acoustic microbubble propulsion, train-like assembly and cargo transport
Source: Nat Commun. 2023 Aug 5;14:4705. doi: 10.1038/s41467-023-40387-7 (PMC10404234; doi:10.1038/s41467-023-40387-7)
Supplement: Supplementary file 15 — Description of Supplementary information files [file 41467_2023_40387_MOESM15_ESM.pdf]

Description of Additional Supplementary Files

Acoustic microbubble propulsion, train-like assembly and cargo transport

Jakub Janiak, Yuyang Li, Yann Ferry, Alexander A. Doinikov, Daniel Ahmed*

Acoustics Robotics Systems Lab (ARSL), Institute for Robotics and Intelligent Systems, ETH Zurich, Rüschlikon, CH-8803, Switzerland

*Correspondence and requests for materials should be addressed to D.A.

(e-mail: dahmed@ethz.ch)

This PDF file includes:

Legends for Supplementary Videos 1 to 14.

Other supplementary materials for this manuscript include the following:

Supplementary Videos 1 to 14.

Supplementary Video 1. Gas nuclei in the slit region when the piezo electric transducer is activated.

Supplementary Video 2. Microbubbles of different sizes are produced across the slit, with the smallest arising at the centre and larger ones radially across the slit.

Supplementary Video 3. Cross-migration of microbubbles. When the acoustic field is turned off, the ellipsoidal microbubbles revert to spherical shape and are squeezed out of the interstice.

Supplementary Video 4. An ellipsoidal microbubble undergoes a volume mode oscillation.

Supplementary Video 5. A Faraday wave developed at the air/liquid interface of an ellipsoidal microbubble.

Supplementary Video 6. Propulsion of an ellipsoidal microbubble along the interstice in the y-direction.

Supplementary Video 7. Increased power to result in the ellipsoidal microbubbles becoming unstable, behaving erratically or merging with other bubbles.

Supplementary Video 8. Propulsion of the microbubble train over long distances.

Supplementary Video 9. Train-like assembly and propulsion of ellipsoidal microbubbles.

Supplementary Video 10. As the voltage of the piezoelectric transducer increases, the interparticle distance between adjacent microbubbles decreases.

Supplementary Video 11. Trapping of microparticle between members of the bubble microtrain.

Supplementary Video 12. Cargo transport: trapping and transport behaviour of the bubble train Trapping of microparticle between members of the bubble microtrain.

Supplementary Video 13. The acoustic streamlines produced by the oscillation of discoidal microbubbles.

Supplementary Video 14. The trapping, transport, and release of superparamagnetic microparticles in gels.
