# Supplementary material for: Acoustic microbubble propulsion, train-like assembly and cargo transport
Source: Nat Commun. 2023 Aug 5;14:4705. doi: 10.1038/s41467-023-40387-7 (PMC10404234; doi:10.1038/s41467-023-40387-7)
Supplement: Supplementary file 16 — Supplementary information [file 41467_2023_40387_MOESM16_ESM.pdf]

Supplementary information

Acoustic microbubble propulsion, train-like assembly and cargo transport

Jakub Janiak, Yuyang Li, Yann Ferry, Alexander A. Doinikov, Daniel Ahmed*

Acoustics Robotics Systems Lab (ARSL), Institute for Robotics and Intelligent Systems, ETH Zurich, Rüschlikon, CH-8803, Switzerland

*Correspondence and requests for materials should be addressed to D.A.

(e-mail: dahmed@ethz.ch)

This PDF file includes:

Supplementary Notes 1 to 14

Supplementary Figures S1 to S13

Supplementary Table S1

Supplementary References

1. Acoustic radiation force on a gas bubble (a solid particle) in the acoustic field generated by two cylinders

It is assumed that there are two closely spaced cylinders surrounded by a liquid (Fig. 1a). The bigger cylinder vibrates radially, generating an acoustic field in the liquid. The smaller cylinder scatters the acoustic field generated by the bigger cylinder. Figure 1b shows two systems of polar coordinates originated at the centers of the cylinders. These coordinate systems are used in calculations.

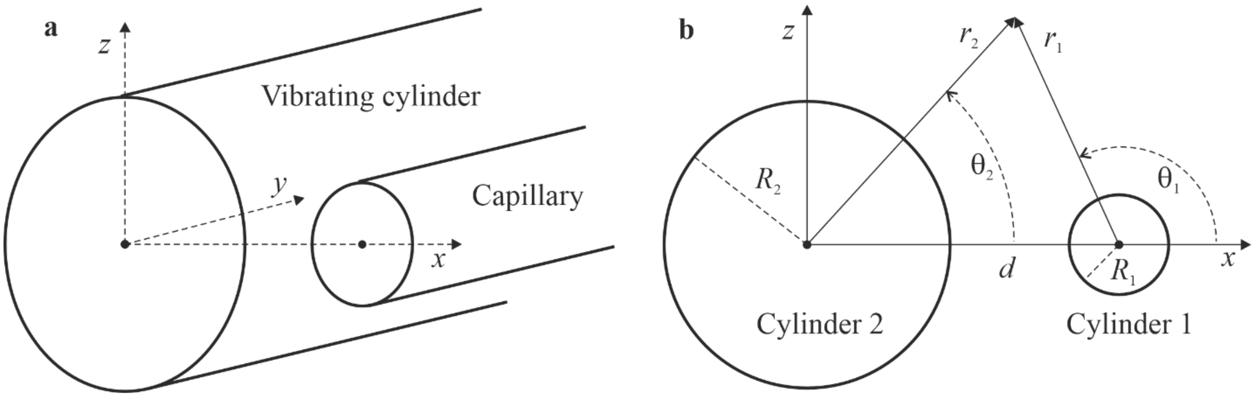

Fig. 1. a, Two closely spaced cylinders surrounded by a liquid. The acoustic field is generated by radial vibrations of the bigger cylinder. **b**, Coordinate systems used in calculations.

1.1. *Acoustic field generated by two cylinders*

We assume that the first-order liquid velocity is represented by $\boldsymbol{v} = \nabla\varphi$, where φ is the velocity potential, and the time dependence is $\exp(-i\omega t)$, where ω is the angular frequency. Then φ should obey the Helmholtz equation [1],

$$\nabla^2\varphi + k^2\varphi = 0, \tag{1.1}$$

where $k = \omega/c$ is the wavenumber and c is the speed of sound in the liquid.

The total potential generated by both cylinders is represented by

$$\varphi = \varphi_1 + \varphi_2, \tag{1.2}$$

where φ_j is the potential of the scattered wave produced by the *j*th cylinder. φ_j, satisfying Eq. (1.1), is given by [2]

$$\varphi_j = e^{-i\omega t}\sum_{n=-\infty}^{\infty} a_n^{(j)} H_n^{(1)}(kr_j) e^{in\theta_j} = e^{-i\omega t}\sum_{n=-\infty}^{\infty} b_n^{(j)} J_n(kr_{3-j}) e^{in\theta_{3-j}} , \tag{1.3}$$

where $a_n^{(j)}$ and $b_n^{(j)}$ are constants, J_n is the Bessel function of the first kind, $H_n^{(1)}$ is the Hankel function of the first kind, the first sum gives φ_j in the coordinates of the *j*th cylinder and the second sum gives φ_j in the coordinates of the other cylinder. We need both expressions in order to satisfy boundary conditions at the surfaces of both cylinders.

With the help of Eqs. (A5) and (A6) from Appendix A, we express $b_n^{(j)}$ in terms of $a_n^{(j)}$,

$$b_n^{(1)} = \sum_{m=-\infty}^{\infty} (-1)^{n+m} H_{m-n}^{(1)}(kd) a_m^{(1)} , \tag{1.4}$$

$$b_n^{(2)} = \sum_{m=-\infty}^{\infty} H_{m-n}^{(1)}(kd) a_m^{(2)} , \tag{1.5}$$

where d is the distance between the centers of the cylinders.

It follows from Eqs. (1.3) – (1.5) that the total potential φ in the coordinates (r_1, θ_1) can be written as

$$\varphi(r_1, \theta_1, t) = e^{-i\omega t}\sum_{n=-\infty}^{\infty} \left[a_n^{(1)} H_n^{(1)}(kr_1) + b_n^{(2)} J_n(kr_1) \right] e^{in\theta_1} , \tag{1.6}$$

and hence the normal component of the liquid velocity is calculated by

$$v_r(r_1, \theta_1, t) = \frac{\partial \varphi}{\partial r_1} = e^{-i\omega t} k \sum_{n=-\infty}^{\infty} \left[a_n^{(1)} H_n^{(1)/}(kr_1) + b_n^{(2)} J_n^{/}(kr_1) \right] e^{in\theta_1} , \tag{1.7}$$

where the prime denotes the derivative with respect to an argument in brackets.

Analogously, in the coordinates (r_2, θ_2), one obtains

$$\varphi(r_2, \theta_2, t) = e^{-i\omega t}\sum_{n=-\infty}^{\infty} \left[a_n^{(2)} H_n^{(1)}(kr_2) + b_n^{(1)} J_n(kr_2) \right] e^{in\theta_2} , \tag{1.8}$$

$$v_r(r_2, \theta_2, t) = e^{-i\omega t} k \sum_{n=-\infty}^{\infty} \left[a_n^{(2)} H_n^{(1)/}(kr_2) + b_n^{(1)} J_n^{/}(kr_2) \right] e^{in\theta_2} . \tag{1.9}$$

To find $a_n^{(j)}$, we apply boundary conditions at the surfaces of the cylinders, which are given by

$$v_r(r_j, \theta_j, t) = V_j e^{-i\omega t} \quad \text{at} \quad r_j = R_j , \tag{1.10}$$

where R_j is the equilibrium radius of the *j*th cylinder and $V_j e^{-i\omega t}$ denotes the normal velocity of the surface of the *j*th cylinder. Assuming that the amplitude of the radial oscillation of the *j*th cylinder is A_j, V_j is calculated by $V_j = -i\omega A_j$.

Substituting Eqs. (1.7) and (1.9) into Eq. (1.10) gives

$$kH_n^{(1)/}(kR_1)a_n^{(1)} + kJ_n^{/}(kR_1)b_n^{(2)} = V_1\delta_{n0}, \tag{1.11}$$

$$kH_n^{(1)/}(kR_2)a_n^{(2)} + kJ_n^{/}(kR_2)b_n^{(1)} = V_2\delta_{n0}, \tag{1.12}$$

where δ_{nm} is the Kronecker delta. Substitution of Eqs. (1.4) and (1.5) into Eqs. (1.11) and (1.12) yields

$$kH_n^{(1)/}(kR_1)a_n^{(1)} + kJ_n^{/}(kR_1)\sum_{m=-\infty}^{\infty} H_{m-n}^{(1)}(kd)a_m^{(2)} = V_1\delta_{n0}, \tag{1.13}$$

$$kH_n^{(1)/}(kR_2)a_n^{(2)} + kJ_n^{/}(kR_2)\sum_{m=-\infty}^{\infty} (-1)^{n+m} H_{m-n}^{(1)}(kd)a_m^{(1)} = V_2\delta_{n0}. \tag{1.14}$$

From Eq. (1.14), one obtains $a_n^{(2)}$ in terms of $a_n^{(1)}$ as

$$a_n^{(2)} = \frac{V_2\delta_{n0}}{kH_n^{(1)/}(kR_2)} - \frac{(-1)^n J_n^{/}(kR_2)}{H_n^{(1)/}(kR_2)}\sum_{s=-\infty}^{\infty}(-1)^s H_{s-n}^{(1)}(kd)a_s^{(1)}. \tag{1.15}$$

Substituting Eq. (1.15) into Eq. (1.13) results in

$$\sum_{m=-\infty}^{\infty} A_{nm}a_m^{(1)} = B_n, \tag{1.16}$$

where

$$A_{nm} = H_n^{(1)/}(kR_1)\delta_{nm} - J_n^{/}(kR_1)(-1)^m \sum_{s=-\infty}^{\infty}\frac{(-1)^s H_{s-n}^{(1)}(kd)H_{m-s}^{(1)}(kd)J_s^{/}(kR_2)}{H_s^{(1)/}(kR_2)}, \tag{1.17}$$

$$B_n = \frac{\delta_{n0}}{k}V_1 + \frac{(-1)^n J_n^{/}(kR_1)H_n^{(1)}(kd)}{kH_1^{(1)}(kR_2)}V_2. \tag{1.18}$$

As one can see, Eq. (1.16) is an infinite system of equations in the unknowns $a_m^{(1)}$. However, the scattering coefficients $a_m^{(1)}$ should decrease with increasing *m*. Therefore, we can truncate system (1.16) setting that *n* and *m* vary from – *N* to *N*. As a result, we will obtain a system of $2N+1$ equations,

$$\sum_{m=-N}^{N} A_{nm}a_m^{(1)} = B_n, \quad n = -N, -N+1, ..., N-1, N. \tag{1.19}$$

In Eq. (1.15), we can also set the limits of the sum to $-N$ and N and then use this equation to calculate $a_n^{(2)}$ for n varying from $-N$ to N.

The liquid pressure at the point (x,z) is calculated by

$$p(x,z,t) = -\rho_0 \frac{\partial \varphi}{\partial t} = i\omega\rho_0 e^{-i\omega t} \sum_{n=-\infty}^{\infty} \left[a_n^{(1)} H_n^{(1)}(kr_1) e^{in\theta_1} + a_n^{(2)} H_n^{(1)}(kr_2) e^{in\theta_2} \right], \qquad (1.20)$$

where ρ_0 is the equilibrium liquid density and the polar coordinates are expressed in terms of the Cartesian coordinates as follows:

$$r_1 = \sqrt{(x-d)^2 + z^2}, \quad \theta_1 = \begin{cases} \arcsin(z/r_1) \text{ if } x \geq d \\ \pi - \arcsin(z/r_1) \text{ if } x < d, z \geq 0 \\ -\pi - \arcsin(z/r_1) \text{ if } x < d, z < 0 \end{cases}, \qquad (1.21)$$

$$r_2 = \sqrt{x^2 + z^2}, \quad \theta_2 = \begin{cases} \arcsin(z/r_2) \text{ if } x \geq 0 \\ \pi - \arcsin(z/r_2) \text{ if } x < 0, z \geq 0 \\ -\pi - \arcsin(z/r_2) \text{ if } x < 0, z < 0 \end{cases}. \qquad (1.22)$$

Note that by definition the values of arcsin lie in the range $-\pi/2$ to $\pi/2$.

1.2. *Radiation force on a gas bubble in the acoustic field of two cylinders*

Let us assume that there is a gas bubble in the liquid surrounding the cylinders. The bubble will experience acoustic radiation force that is calculated by [3]

$$\boldsymbol{F}_b = -\langle V_b \nabla p \rangle, \qquad (1.23)$$

where V_b is the time-varying volume of the bubble and $\langle \ \rangle$ means the time average.

Let us assume that the bubble undergoes a small-amplitude radial oscillation so that its radius is represented by

$$R(t) = R_0 + a(t), \quad a << R_0, \qquad (1.24)$$

where R_0 is the equilibrium radius of the bubble and $a(t)$ is the amplitude of the bubble oscillation. Then we can write

$$V_b = \frac{4}{3}\pi R^3 \approx \frac{4}{3}\pi R_0^3 \left(1 + \frac{3a}{R_0}\right). \qquad (1.25)$$

Substitution of Eq. (1.25) into Eq. (1.23) gives

$$\boldsymbol{F}_b = -4\pi R_0^2 \langle a \nabla p \rangle. \qquad (1.26)$$

To find $a(t)$, we use the Rayleigh-Plesset equation [4,5],

$$R\ddot{R} + \frac{3}{2}\dot{R}^2 = \frac{1}{\rho_0}\left[P_g - \frac{2\sigma}{R} - \frac{4\eta\dot{R}}{R} - P_0 - p(x,y,t) \right], \tag{1.27}$$

where the overdot means the time derivative, P_g is the gas pressure inside the bubble, σ is the surface tension, η is the dynamic liquid viscosity, and P_0 is the hydrostatic pressure in the liquid.

Assuming that P_g obeys the adiabatic law with the exponent γ, one obtains

$$P_g = P_{g0}\left(\frac{R_0}{R}\right)^{3\gamma} \approx P_{g0}\left(1 - \frac{3\gamma a}{R_0}\right), \tag{1.28}$$

where P_{g0} denotes the equilibrium gas pressure.

Linearizing Eq. (1.27) in view of the smallness of $a(t)$ and using Eq. (1.28), one obtains

$$P_{g0} = P_0 + \frac{2\sigma}{R_0}, \tag{1.29}$$

$$\ddot{a} + \omega\delta\dot{a} + \omega_0^2 a = -\frac{p(x,z,t)}{\rho_0 R_0}, \tag{1.30}$$

where ω_0 is the angular resonance frequency of the bubble, given by

$$\omega_0 = \frac{1}{R_0}\sqrt{\frac{3\gamma P_0}{\rho_0} + \frac{2(3\gamma - 1)\sigma}{\rho_0 R_0}}, \tag{1.31}$$

and δ is the viscous damping constant, given by

$$\delta = \frac{4\eta}{\omega\rho_0 R_0^2}. \tag{1.32}$$

Since $a(t) \sim \exp(-i\omega t)$, Eq. (1.30) gives

$$a(t) = \frac{p(x,z,t)}{\rho_0 R_0(\omega^2 - \omega_0^2 + i\omega^2\delta)}. \tag{1.33}$$

Substitution of Eq. (1.33) into Eq. (1.26) yields

$$\boldsymbol{F}_b = \frac{2\pi R_0}{\rho_0}\mathrm{Re}\left\{\frac{p^*\nabla p}{\omega_0^2 - \omega^2 + i\omega^2\delta}\right\}, \tag{1.34}$$

where Re means “the real part of” and the asterisk denotes the complex conjugate. On substitution of Eq. (1.20) into Eq. (1.34), one obtains

$$\boldsymbol{F}_b = 2\pi\rho_0 R_0 \omega^2 \,\mathrm{Re}\left\{ \frac{1}{\omega_0^2 - \omega^2 + i\omega^2\delta} \sum_{n=-\infty}^{\infty} \left[a_n^{(1)*} H_n^{(1)*}(kr_1) e^{-in\theta_1} + a_n^{(2)*} H_n^{(1)*}(kr_2) e^{-in\theta_2} \right] \right.$$

$$\times \left\{ \sum_{m=-\infty}^{\infty} \left[\boldsymbol{e}_{r1} k a_m^{(1)} H_m^{(1)/}(kr_1) e^{im\theta_1} + \boldsymbol{e}_{r2} k a_m^{(2)} H_m^{(1)/}(kr_2) e^{im\theta_2} \right] \right.$$

$$\left. \left. + \sum_{m=-\infty}^{\infty} \left[\boldsymbol{e}_{\theta 1} \frac{im}{r_1} a_m^{(1)} H_m^{(1)}(kr_1) e^{im\theta_1} + \boldsymbol{e}_{\theta 2} \frac{im}{r_2} a_m^{(2)} H_m^{(1)}(kr_2) e^{im\theta_2} \right] \right\} \right\}. \tag{1.35}$$

The unit vectors of the polar coordinates, $\boldsymbol{e}_{rj}$ and $\boldsymbol{e}_{\theta j}$, are defined by

$$\boldsymbol{e}_{rj} = \boldsymbol{e}_x \cos\theta_j + \boldsymbol{e}_z \sin\theta_j, \quad \boldsymbol{e}_{\theta j} = -\boldsymbol{e}_x \sin\theta_j + \boldsymbol{e}_z \cos\theta_j. \tag{1.36}$$

Accordingly, the radiation force on the bubble located at the point (x,z) can be represented by

$$\boldsymbol{F}_b = F_{bx}\boldsymbol{e}_x + F_{bz}\boldsymbol{e}_z, \tag{1.37}$$

where F_{bx} and F_{bz} are calculated by

$$F_{bx} = 2\pi\rho_0 k R_0 \,\mathrm{Re}\left\{ \frac{G_x(x,z)}{\omega_0^2/\omega^2 - 1 + i\delta} \right\}, \tag{1.38}$$

$$F_{bz} = 2\pi\rho_0 k R_0 \,\mathrm{Re}\left\{ \frac{G_z(x,z)}{\omega_0^2/\omega^2 - 1 + i\delta} \right\}. \tag{1.39}$$

The functions appearing in Eqs. (1.38) and (1.39) are given by

$$G_x(x,z) = \sum_{n,m=-\infty}^{\infty} F_n^*(r_1,\theta_1,r_2,\theta_2) D_m(r_1,\theta_1,r_2,\theta_2), \tag{1.40}$$

$$G_z(x,z) = \sum_{n,m=-\infty}^{\infty} F_n^*(r_1,\theta_1,r_2,\theta_2) E_m(r_1,\theta_1,r_2,\theta_2), \tag{1.41}$$

$$F_n(r_1,\theta_1,r_2,\theta_2) = a_n^{(1)} H_n^{(1)}(kr_1) e^{in\theta_1} + a_n^{(2)} H_n^{(1)}(kr_2) e^{in\theta_2}, \tag{1.42}$$

$$D_m(r_1,\theta_1,r_2,\theta_2) = a_m^{(1)} e^{im\theta_1} \left(\cos\theta_1 H_m^{(1)/}(kr_1) - \sin\theta_1 \frac{imH_m^{(1)}(kr_1)}{kr_1} \right)$$

$$+ a_m^{(2)} e^{im\theta_2} \left(\cos\theta_2 H_m^{(1)/}(kr_2) - \sin\theta_2 \frac{imH_m^{(1)}(kr_2)}{kr_2} \right), \tag{1.43}$$

$$E_m(r_1,\theta_1,r_2,\theta_2) = a_m^{(1)} e^{im\theta_1} \left(\sin\theta_1 H_m^{(1)/}(kr_1) + \cos\theta_1 \frac{imH_m^{(1)}(kr_1)}{kr_1} \right)$$

$$+a_m^{(2)}e^{im\theta_2}\left(\sin\theta_2 H_m^{(1)/}(kr_2)+\cos\theta_2\frac{imH_m^{(1)}(kr_2)}{kr_2}\right). \quad (1.44)$$

Here r_j and θ_j are calculated by Eqs. (1.21) and (1.22).

In numerical calculations, the limits of the sums in Eqs. (1.40) and (1.41) should be set to – *N* and *N*.

1.3. *Radiation force on a solid particle in the acoustic field of two cylinders*

According to Gor'kov's theory [6], the acoustic radiation force experienced by a solid particle in the acoustic field generated by the two cylinders is calculated by

$$\boldsymbol{F}_p^{(c)}=-\nabla U_c, \quad (1.45)$$

where the force potential U_c is defined by

$$U_c=2\pi R_p^3\rho_0\left(\frac{\langle p^2\rangle}{3\rho_0^2c^2}f_1-\frac{\langle \boldsymbol{v}^2\rangle}{2}f_2\right). \quad (1.46)$$

Here $f_1=1-c^2\rho_0/(c_p^2\rho_p)$, $f_2=2(\rho_p-\rho_0)/(2\rho_p+\rho_0)$, R_p is the radius of the particle, ρ_p is the density of the particle, and c_p is the speed of sound in the particle.

Substituting p and $\boldsymbol{v}$ into Eq. (1.46) and using Eq. (1.36), one obtains the *x* and *z* components of the radiation force as follows:

$$F_{px}^{(c)}=2\pi\rho_0R_p^3\left[\frac{\omega^2 f_1}{3c^2}H_x^{(c)}(x,z)-\frac{f_2}{2}I_x^{(c)}(x,z)\right], \quad (1.47)$$

$$F_{pz}^{(c)}=2\pi\rho_0R_p^3\left[\frac{\omega^2 f_1}{3c^2}H_z^{(c)}(x,z)-\frac{f_2}{2}I_z^{(c)}(x,z)\right], \quad (1.48)$$

where

$$H_x^{(c)}(x,z)=-k\,\mathrm{Re}\sum_{n,m=-\infty}^{\infty}F_n^*(r_1,\theta_1,r_2,\theta_2)D_m(r_1,\theta_1,r_2,\theta_2), \quad (1.49)$$

$$I_x^{(c)}(x,z)=-k^3\,\mathrm{Re}\sum_{n,m=-\infty}^{\infty}\left[a_n^{(1)}a_m^{(1)*}G_{nm}^{(x)}(r_1,\theta_1)+a_n^{(2)}a_m^{(2)*}G_{nm}^{(x)}(r_2,\theta_2)+a_n^{(1)}a_m^{(2)*}Q_{nm}^{(x)}(r_1,\theta_1,r_2,\theta_2)\right], \quad (1.50)$$

$$H_z^{(c)}(x,z)=-k\,\mathrm{Re}\sum_{n,m=-\infty}^{\infty}F_n^*(r_1,\theta_1,r_2,\theta_2)E_m(r_1,\theta_1,r_2,\theta_2), \quad (1.51)$$

$$I_z^{(c)}(x,z) = -k^3 \operatorname{Re} \sum_{n,m=-\infty}^{\infty} \left[a_n^{(1)} a_m^{(1)*} G_{nm}^{(z)}(r_1,\theta_1) + a_n^{(2)} a_m^{(2)*} G_{nm}^{(z)}(r_2,\theta_2) + a_n^{(1)} a_m^{(2)*} Q_{nm}^{(z)}(r_1,\theta_1,r_2,\theta_2) \right], \quad (1.52)$$

$$G_{nm}^{(x)}(r_j,\theta_j) = \frac{1}{2} e^{i(n-m)\theta_j} \left[g_{nm}^{(1)}(r_j)\cos\theta_j - g_{nm}^{(2)}(r_j)\sin\theta_j \right], \quad (1.53)$$

$$G_{nm}^{(z)}(r_j,\theta_j) = \frac{1}{2} e^{i(n-m)\theta_j} \left[g_{nm}^{(1)}(r_j)\sin\theta_j + g_{nm}^{(2)}(r_j)\cos\theta_j \right], \quad (1.54)$$

$$\begin{aligned}
Q_{nm}^{(x)}(r_1,\theta_1,r_2,\theta_2) = e^{in\theta_1} e^{-im\theta_2} \Big\{ & \cos(\theta_1-\theta_2) \left[q_{nm}^{(1)}(r_1,r_2)\cos\theta_1 + q_{mn}^{(1)*}(r_2,r_1)\cos\theta_2 \right] \\
& -\sin(\theta_1-\theta_2) \left[q_{nm}^{(2)}(r_1,r_2)\cos\theta_1 - q_{mn}^{(2)*}(r_2,r_1)\cos\theta_2 \right] \\
& -q_{nm}^{(3)}(r_1,r_2) \left\{ \frac{\sin\theta_1}{kr_1} \left[in\cos(\theta_1-\theta_2) - \sin(\theta_1-\theta_2) \right] - \frac{\sin\theta_2}{kr_2} \left[im\cos(\theta_1-\theta_2) - \sin(\theta_1-\theta_2) \right] \right\} \\
& +q_{nm}^{(4)}(r_1,r_2) \left\{ \frac{\sin\theta_1}{kr_1} \left[in\sin(\theta_1-\theta_2) + \cos(\theta_1-\theta_2) \right] - \frac{\sin\theta_2}{kr_2} \left[im\sin(\theta_1-\theta_2) + \cos(\theta_1-\theta_2) \right] \right\} \Big\}, \quad (1.55)
\end{aligned}$$

$$\begin{aligned}
Q_{nm}^{(z)}(r_1,\theta_1,r_2,\theta_2) = e^{in\theta_1} e^{-im\theta_2} \Big\{ & \cos(\theta_1-\theta_2) \left[q_{nm}^{(1)}(r_1,r_2)\sin\theta_1 + q_{mn}^{(1)*}(r_2,r_1)\sin\theta_2 \right] \\
& -\sin(\theta_1-\theta_2) \left[q_{nm}^{(2)}(r_1,r_2)\sin\theta_1 - q_{mn}^{(2)*}(r_2,r_1)\sin\theta_2 \right] \\
& +q_{nm}^{(3)}(r_1,r_2) \left\{ \frac{\cos\theta_1}{kr_1} \left[in\cos(\theta_1-\theta_2) - \sin(\theta_1-\theta_2) \right] - \frac{\cos\theta_2}{kr_2} \left[im\cos(\theta_1-\theta_2) - \sin(\theta_1-\theta_2) \right] \right\} \\
& -q_{nm}^{(4)}(r_1,r_2) \left\{ \frac{\cos\theta_1}{kr_1} \left[in\sin\theta_1-\theta_2) + \cos(\theta_1-\theta_2) \right] - \frac{\cos\theta_2}{kr_2} \left[im\sin(\theta_1-\theta_2) + \cos(\theta_1-\theta_2) \right] \right\} \Big\}, \quad (1.56)
\end{aligned}$$

$$\begin{aligned}
g_{nm}^{(1)}(r_j) = {} & H_n^{(1)''}(kr_j) H_m^{(1)'*}(kr_j) + H_n^{(1)'}(kr_j) H_m^{(1)''*}(kr_j) \\
& + \frac{nm}{(kr_j)^3} \left\{ H_n^{(1)}(kr_j) \left[kr_j H_m^{(1)'*}(kr_j) - H_m^{(1)*}(kr_j) \right] + H_m^{(1)*}(kr_j) \left[kr_j H_n^{(1)'}(kr_j) - H_n^{(1)}(kr_j) \right] \right\}, \quad (1.57)
\end{aligned}$$

$$g_{nm}^{(2)}(r_j) = \frac{i(n-m)}{(kr_j)^3} \left[(kr_j)^2 H_n^{(1)'}(kr_j) H_m^{(1)'*}(kr_j) + nm H_n^{(1)}(kr_j) H_m^{(1)*}(kr_j) \right], \quad (1.58)$$

$$q_{nm}^{(1)}(r_1,r_2) = H_n^{(1)''}(kr_1) H_m^{(1)'*}(kr_2) + \frac{nm}{k^2 r_1 r_2} H_n^{(1)'}(kr_1) H_m^{(1)*}(kr_2), \quad (1.59)$$

$$q_{nm}^{(2)}(r_1,r_2)=\frac{in}{(kr_1)^2}\left[kr_1H_n^{(1)/}(kr_1)-H_n^{(1)}(kr_1)\right]H_m^{(1)/*}(kr_2)\,, \tag{1.60}$$

$$q_{nm}^{(3)}(r_1,r_2)=H_n^{(1)/}(kr_1)H_m^{(1)/*}(kr_2)+\frac{nm}{k^2r_1r_2}H_n^{(1)}(kr_1)H_m^{(1)*}(kr_2)\,, \tag{1.61}$$

$$q_{nm}^{(4)}(r_1,r_2)=\frac{in}{kr_1}H_n^{(1)}(kr_1)H_m^{(1)/*}(kr_2)+\frac{im}{kr_2}H_n^{(1)/}(kr_1)H_m^{(1)*}(kr_2)\,. \tag{1.62}$$

Here r_j and θ_j are calculated by Eqs. (1.21) and (1.22).

In numerical calculations, the limits of the sums in Eqs. (1.49) – (1.52) should be set to –*N* and *N*.

2. Acoustic radiation force on a solid particle in the acoustic field generated by two oscillating gas bubbles

We consider two gas bubbles undergoing axisymmetric oscillations. The bubbles are assumed to be spherical at rest. In this calculation, we use two systems of spherical coordinates originated at the equilibrium centers of the bubbles (Fig. 2).

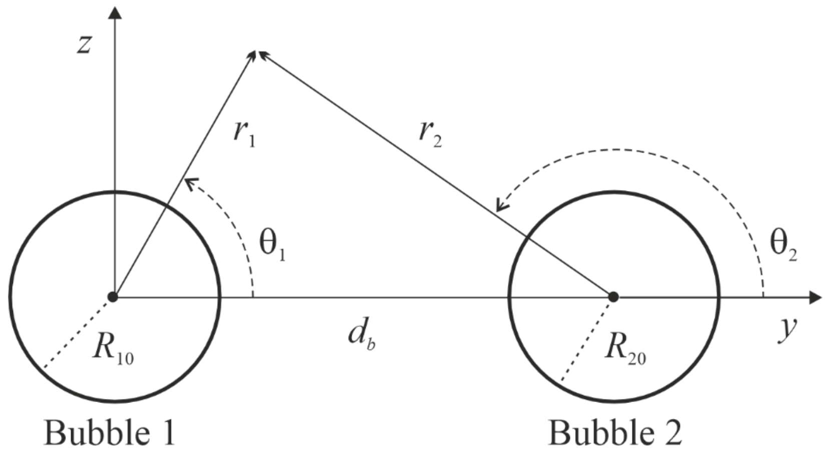

Fig. 2. Spherical coordinate systems used in the calculation of the radiation force on a solid particle in the acoustic field generated by two bubbles.

2.1. *Acoustic field generated by two bubbles*

The surface of the *j*th bubble ($j = 1, 2$) can be represented as

$$r_s^{(j)} = R_{j0} + e^{-i\omega t} \sum_{n=0}^{\infty} s_n^{(j)} P_n(\mu_j), \tag{2.1}$$

where R_{j0} is the equilibrium radius of the *j*th bubble, P_n is the Legendre polynomial of degree *n*, $\mu_j = \cos\theta_j$, and $s_n^{(j)}$ is the complex amplitude of the *n*th oscillation mode of the *j*th bubble. We assume that $s_n^{(j)}$ is known, i.e., the value of $s_n^{(j)}$ is an input parameter in our theory.

The total first-order liquid velocity generated by both bubbles is given by

$$\boldsymbol{v}_b = \boldsymbol{v}_b^{(1)} + \boldsymbol{v}_b^{(2)}, \tag{2.2}$$

where $\boldsymbol{v}_b^{(j)}$ is the first-order liquid velocity generated by the *j*th bubble.

$\boldsymbol{v}_b^{(j)}$ can be represented by

$$\boldsymbol{v}_b^{(j)} = \nabla \varphi_b^{(j)} , \tag{2.3}$$

where $\varphi_b^{(j)}$ is the scalar velocity potentials of the *j*th bubble. Assuming that the size of the bubbles is small compared to the acoustic wavelength, we can ignore the liquid compressibility and represent $\varphi_b^{(j)}$ as

$$\varphi_b^{(j)} = e^{-i\omega t} \sum_{n=0}^{\infty} c_n^{(j)} \left(\frac{R_{j0}}{r_j} \right)^{n+1} P_n(\mu_j) , \tag{2.4}$$

where $c_n^{(j)}$ are constant coefficients to be determined by boundary conditions at the bubbles' surfaces.

From Eq. (2.4), one obtains

$$v_{br}^{(j)} = -\frac{e^{-i\omega t}}{r_j} \sum_{n=0}^{\infty} (n+1) c_n^{(j)} \left(\frac{R_{j0}}{r_j} \right)^{n+1} P_n(\mu_j) , \tag{2.5}$$

$$v_{b\theta}^{(j)} = \frac{e^{-i\omega t}}{r_j} \sum_{n=1}^{\infty} c_n^{(j)} \left(\frac{R_{j0}}{r_j} \right)^{n+1} P_n^1(\mu_j) , \tag{2.6}$$

where P_n^1 is the associated Legendre polynomial of the first order and degree *n*.

The total first-order liquid pressure generated by the bubbles is calculated by

$$p_b = i\omega\rho_0 \left(\varphi_b^{(1)} + \varphi_b^{(2)} \right) , \tag{2.7}$$

where ρ_0 is the liquid density.

The boundary condition for the liquid velocity requires that the normal component of $\boldsymbol{v}_b$ at $r_j = R_{j0}$ be equal to the normal component of the surface velocity of the *j*th bubble,

$$v_{br} \,|_{r_j = R_{j0}} = \frac{dr_s^{(j)}}{dt} = -i\omega e^{-i\omega t} \sum_{n=0}^{\infty} s_n^{(j)} P_n(\mu_j) . \tag{2.8}$$

To apply this boundary condition, we transform the expressions of $\varphi_b^{(1)}$ and $\varphi_b^{(2)}$ by the following identities [7,8]:

$$\frac{P_n(\mu_1)}{r_1^{n+1}} = \frac{1}{d_b^{n+1}} \sum_{m=0}^{\infty} (-1)^m C_{nm} \left(\frac{r_2}{d_b} \right)^m P_m(\mu_2) , \tag{2.9}$$

$$\frac{P_n(\mu_2)}{r_2^{n+1}} = \frac{(-1)^n}{d^{n+1}} \sum_{m=0}^{\infty} C_{nm} \left(\frac{r_1}{d} \right)^m P_m(\mu_1) , \tag{2.10}$$

where $C_{nm} = (n+m)!/(n!m!)$ and d_b is the distance between the equilibrium centers of the bubbles. Note that Eqs. (2.9) and (2.10) are valid for $r_{1,2} < d_b$.

Substituting Eq. (2.9) into Eq. (2.4) at $j = 1$ and Eq. (2.10) into Eq. (2.4) at $j = 2$, one obtains

$$\varphi_b^{(1)}(r_2,\theta_2) = e^{-i\omega t} \sum_{n,m=0}^{\infty} c_n^{(1)} \xi_1^{n+1} (-1)^m C_{nm} \left(\frac{r_2}{d_b} \right)^m P_m(\mu_2), \tag{2.11}$$

$$\varphi_b^{(2)}(r_1,\theta_1) = e^{-i\omega t} \sum_{n,m=0}^{\infty} (-1)^n c_n^{(2)} \xi_2^{n+1} C_{nm} \left(\frac{r_1}{d_b} \right)^m P_m(\mu_1), \tag{2.12}$$

where $\xi_j = R_{j0}/d_b$. Equation (2.11) gives $\varphi_b^{(1)}$ in the coordinates (r_2, θ_2) near the surface of bubble 2, while Eq. (2.12) gives $\varphi_b^{(2)}$ in the coordinates (r_1, θ_1) near the surface of bubble 1, i.e., these equations can be used in the corresponding boundary conditions.

The normal component of the liquid velocity at the surface of bubble 1 is given by

$$v_{br}(R_{10},\theta_1) = \frac{e^{-i\omega t}}{R_{10}} \sum_{n=0}^{\infty} \left[-(n+1)c_n^{(1)} + n\xi_1^n \sum_{m=0}^{\infty} (-1)^m C_{nm} \xi_2^{m+1} c_m^{(2)} \right] P_n(\mu_1). \tag{2.13}$$

Substitution of Eq. (2.13) into Eq. (2.8) yields

$$(n+1)c_n^{(1)} - n\xi_1^n \sum_{m=0}^{\infty} (-1)^m C_{nm} \xi_2^{m+1} c_m^{(2)} = i\omega R_{10} s_n^{(1)}, \ n \ge 0. \tag{2.14}$$

Doing similar calculations for bubble 2, one obtains

$$(n+1)c_n^{(2)} - (-1)^n n\xi_2^n \sum_{m=0}^{\infty} C_{nm} \xi_1^{m+1} c_m^{(1)} = i\omega R_{20} s_n^{(2)}, \ n \ge 0. \tag{2.15}$$

It follows from Eqs. (2.14) and (2.15) that $c_0^{(1)}$ and $c_0^{(2)}$ are calculated by

$$c_0^{(j)} = i\omega R_{j0} s_0^{(j)}, \ j = 1,2 \ . \tag{2.16}$$

For $n \ge 1$, substituting Eq. (2.16) into Eqs. (2.14) and (2.15), one obtains

$$(n+1)c_n^{(1)} = n\xi_1^n \sum_{m=1}^{\infty} (-1)^m C_{nm} \xi_2^{m+1} c_m^{(2)} + i\omega R_{10} s_n^{(1)} + i\omega R_{20} n\xi_1^n \xi_2 s_0^{(2)}, \ n \ge 1, \tag{2.17}$$

$$(n+1)c_n^{(2)} = (-1)^n n\xi_2^n \sum_{m=1}^{\infty} C_{nm} \xi_1^{m+1} c_m^{(1)} + i\omega R_{20} s_n^{(2)} + i\omega R_{10} (-1)^n n\xi_2^n \xi_1 s_0^{(1)}, \ n \ge 1. \tag{2.18}$$

Substituting $c_n^{(2)}$ from Eq. (2.18) into Eq. (2.17), one gets

$$\sum_{m=1}^{\infty} A_{nm}^{(1)} c_m^{(1)} = B_n^{(1)}, \ n \ge 1, \tag{2.19}$$

where

$$A_{nm}^{(1)} = (n+1)\delta_{nm} - D_{nm}^{(1)}, \tag{2.20}$$

$$B_n^{(1)} = i\omega R_{10}\left(s_n^{(1)} + D_{n0}^{(1)} s_0^{(1)}\right) + i\omega R_{20} n\xi_1^n \xi_2 \left[s_0^{(2)} + \sum_{k=0}^{\infty} \frac{(-1)^k C_{nk} \xi_2^k}{k+1} s_k^{(2)} \right], \tag{2.21}$$

$$D_{nm}^{(1)} = n\xi_1^{n+m+1} \sum_{k=1}^{\infty} \frac{k C_{nk} C_{mk} \xi_2^{2k+1}}{k+1}, \tag{2.22}$$

and δ_{nm} is the Kronecker delta.

As one can see, Eq. (2.19) is an infinite system of equations in the unknowns $c_m^{(1)}$. However, the coefficients $c_m^{(1)}$ should decrease with increasing *m*. Therefore, we can truncate system (2.19) setting that *n* and *m* vary from 1 to *N*. As a result, we will obtain a system of N equations,

$$\sum_{m=1}^{N} A_{nm}^{(1)} c_m^{(1)} = B_n^{(1)}, \quad n = 1, 2, ..., N-1, N. \tag{2.23}$$

Similarly, for $c_m^{(2)}$, one obtains

$$\sum_{m=1}^{N} A_{nm}^{(2)} c_m^{(2)} = B_n^{(2)}, \quad n = 1, 2, ..., N-1, N, \tag{2.24}$$

where

$$A_{nm}^{(2)} = (n+1)\delta_{nm} - D_{nm}^{(2)}, \tag{2.25}$$

$$B_n^{(2)} = i\omega R_{20}\left(s_n^{(2)} + D_{n0}^{(2)} s_0^{(2)}\right) + i\omega R_{10} (-1)^n n\xi_2^n \xi_1 \left[s_0^{(1)} + \sum_{k=1}^{\infty} \frac{C_{nk} \xi_1^k}{k+1} s_k^{(1)} \right], \tag{2.26}$$

$$D_{nm}^{(2)} = (-1)^{n+m} n\xi_2^{n+m+1} \sum_{k=1}^{\infty} \frac{k C_{nk} C_{mk} \xi_1^{2k+1}}{k+1}. \tag{2.27}$$

Solving systems (2.23) and (2.24) provides $c_n^{(1)}$ and $c_n^{(2)}$ for $n \geq 1$.

2.2. *Radiation force on a solid particle in the acoustic field of two bubbles*

According to Gor'kov's theory [6], the acoustic radiation force experienced by a solid particle in the acoustic field generated by the two oscillating bubbles is calculated by

$$\boldsymbol{F}_p^{(b)} = -\nabla U_b, \tag{2.28}$$

where the force potential U_b is defined by

$$U_b = 2\pi\rho_0 R_p^3 \left(\frac{\langle p_b^2 \rangle}{3\rho_0^2 c^2} f_1 - \frac{\langle \boldsymbol{v}_b^2 \rangle}{2} f_2 \right). \quad (2.29)$$

Here $f_1 = 1 - c^2\rho_0 / \left(c_p^2 \rho_p\right)$, $f_2 = 2(\rho_p - \rho_0)/(2\rho_p + \rho_0)$, R_p is the radius of the particle, ρ_p is the density of the particle, c_p is the speed of sound in the particle, *c* is the speed of sound in the liquid, and $\langle \ \rangle$ denotes the time average.

We assume that the particle is located at a point with Cartesian coordinates (y, z) (Fig. 2). The coordinate vectors of the *j*th bubble are expressed in terms of the coordinate vectors of the Cartesian system shown in Fig. 2 as follows:

$$\boldsymbol{e}_{rj} = \boldsymbol{e}_y \cos\theta_j + \boldsymbol{e}_z \sin\theta_j, \quad \boldsymbol{e}_{\theta j} = -\boldsymbol{e}_y \sin\theta_j + \boldsymbol{e}_z \cos\theta_j. \quad (2.30)$$

The spherical coordinates of the particle are expressed in terms of the Cartesian coordinates by

$$r_1 = \sqrt{y^2 + z^2}, \quad \theta_1 = \begin{cases} \arcsin(z/r_1) \text{ if } y \ge 0 \\ \pi - \arcsin(z/r_1) \text{ if } y < 0, z \ge 0 \\ -\pi - \arcsin(z/r_1) \text{ if } y < 0, z < 0 \end{cases}, \quad (2.31)$$

$$r_2 = \sqrt{(y - d_b)^2 + z^2}, \quad \theta_2 = \begin{cases} \arcsin(z/r_2) \text{ if } y \ge d_b \\ \pi - \arcsin(z/r_2) \text{ if } y < d_b, z \ge 0 \\ -\pi - \arcsin(z/r_2) \text{ if } y < d_b, z < 0 \end{cases}. \quad (2.32)$$

Note that by definition the values of arcsin lie in the range $-\pi/2$ to $\pi/2$.

Substituting $\boldsymbol{v}_b$ and p_b into Eq. (2.29), one obtains the *y* and *z* components of the radiation force as follows:

$$F_{py}^{(b)} = 2\pi\rho_0 R_p^3 \left[\frac{\omega^2 f_1}{3c^2} H_y^{(b)}(y, z) - \frac{f_2}{2} I_y^{(b)}(y, z) \right], \quad (2.33)$$

$$F_{pz}^{(b)} = 2\pi\rho_0 R_p^3 \left[\frac{\omega^2 f_1}{3c^2} H_z^{(b)}(y, z) - \frac{f_2}{2} I_z^{(b)}(y, z) \right], \quad (2.34)$$

where

$$H_y^{(b)}(y, z) = \operatorname{Re} \sum_{n,m=0}^{N} X_n(r_1, \theta_1, r_2, \theta_2) S_m^*(r_1, \theta_1, r_2, \theta_2), \quad (2.35)$$

$$I_y^{(b)}(y, z) = \operatorname{Re} \sum_{n,m=0}^{N} \left\{ X_m^*(r_1, \theta_1, r_2, \theta_2)[\alpha_n^{(1)}(r_1, \theta_1) + \alpha_n^{(2)}(r_2, \theta_2)] + Y_m^*[\beta_n^{(1)}(r_1, \theta_1) + \beta_n^{(2)}(r_2, \theta_2)] \right\}, \quad (2.36)$$

$$H_z^{(b)}(y,z) = \mathrm{Re}\sum_{n,m=0}^{N} Y_n(r_1,\theta_1,r_2,\theta_2) S_m^*(r_1,\theta_1,r_2,\theta_2), \quad (2.37)$$

$$I_z^{(b)}(y,z) = \mathrm{Re}\sum_{n,m=0}^{N} \left\{ X_m^*(r_1,\theta_1,r_2,\theta_2)[\chi_n^{(1)}(r_1,\theta_1) + \chi_n^{(2)}(r_2,\theta_2)] + Y_m^*[\varepsilon_n^{(1)}(r_1,\theta_1) + \varepsilon_n^{(2)}(r_2,\theta_2)] \right\}, \quad (2.38)$$

$$S_m(r_1,\theta_1,r_2,\theta_2) = c_m^{(1)}\left(\frac{R_{10}}{r_1}\right)^{m+1} P_m(\mu_1) + c_m^{(2)}\left(\frac{R_{20}}{r_2}\right)^{m+1} P_m(\mu_2), \quad (2.39)$$

$$X_n(r_1,\theta_1,r_2,\theta_2) = \frac{c_n^{(1)}}{r_1}\left(\frac{R_{10}}{r_1}\right)^{n+1} [(n+1)\cos\theta_1 P_n(\mu_1) + \sin\theta_1 P_n^1(\mu_1)]$$

$$+\frac{c_n^{(2)}}{r_2}\left(\frac{R_{20}}{r_2}\right)^{n+1} [(n+1)\cos\theta_2 P_n(\mu_2) + \sin\theta_2 P_n^1(\mu_2)], \quad (2.40)$$

$$Y_n(r_1,\theta_1,r_2,\theta_2) = \frac{c_n^{(1)}}{r_1}\left(\frac{R_{10}}{r_1}\right)^{n+1} [(n+1)\sin\theta_1 P_n(\mu_1) - \cos\theta_1 P_n^1(\mu_1)]$$

$$+\frac{c_n^{(2)}}{r_2}\left(\frac{R_{20}}{r_2}\right)^{n+1} [(n+1)\sin\theta_2 P_n(\mu_2) - \cos\theta_2 P_n^1(\mu_2)], \quad (2.41)$$

$$\alpha_n^{(j)}(r_j,\theta_j) = \frac{c_n^{(j)}}{r_j^2}\left(\frac{R_{j0}}{r_j}\right)^{n+1} \left\{(n+1)[(n+2)\cos^2\theta_j - (n+1)\sin^2\theta_j]P_n(\mu_j)\right.$$

$$\left. +(2n+3)\cos\theta_j \sin\theta_j P_n^1(\mu_j)\right\}, \quad (2.42)$$

$$\beta_n^{(j)}(r_j,\theta_j) = \frac{c_n^{(j)}}{r_j^2}\left(\frac{R_{j0}}{r_j}\right)^{n+1}$$

$$\times\left\{(n+1)(2n+3)\cos\theta_j \sin\theta_j P_n(\mu_j) + [(n+2)\sin^2\theta_j - (n+1)\cos^2\theta_j]P_n^1(\mu_j)\right\}, \quad (2.43)$$

$$\chi_n^{(j)}(r_j,\theta_j) = \frac{c_n^{(j)}}{r_j^2}\left(\frac{R_{j0}}{r_j}\right)^{n+1} \left\{(n+1)(2n+3)\cos\theta_j \sin\theta_j P_n(\mu_j)\right.$$

$$\left. +[(n+2)\sin^2\theta_j - (n+1)\cos^2\theta_j]P_n^1(\mu_j)\right\}, \quad (2.44)$$

$$\varepsilon_n^{(j)}(r_j,\theta_j) = \frac{c_n^{(j)}}{r_j^2}\left(\frac{R_{j0}}{r_j}\right)^{n+1} \left\{(n+1)[(n+2)\sin^2\theta_j - (n+1)\cos^2\theta_j]P_n(\mu_j)\right.$$

$$\left. -(2n+3)\cos\theta_j \sin\theta_j P_n^1(\mu_j) + \cos\theta_j \sum_{k=1}^{[(n+1)/2]} (2n-4k+3)P_{n-2k+1}(\mu_j)\right\}. \quad (2.45)$$

Here r_j and θ_j are calculated by Eqs. (2.31) and (2.32).

3. Total radiation force on a solid particle in the system under study

The total radiation force on a solid particle in the system under study has two parts. One part is caused by the acoustic field generated by the two cylinders. The other part is caused by the acoustic field generated by the two bubbles, which are between the cylinders. Therefore, in the plane (y,z), which is the plane of observation in our experiments, the components of the total radiation force on a solid particle are given by

$$F_{py} = 2\pi\rho_0 R_p^3 \left[\frac{\omega^2 f_1}{3c^2} H_y(y,z) - \frac{f_2}{2} I_y(y,z) \right], \tag{3.1}$$

$$F_{pz} = 2\pi\rho_0 R_p^3 \left[\frac{\omega^2 f_1}{3c^2} H_z(y,z) - \frac{f_2}{2} I_z(y,z) \right], \tag{3.2}$$

where

$$H_y(y,z) = H_y^{(b)}(y,z), \quad I_y(y,z) = I_y^{(b)}(y,z), \tag{3.3}$$

$$H_z(y,z) = H_z^{(c)}(x,z) + H_z^{(b)}(y,z), \tag{3.4}$$

$$I_z(y,z) = I_z^{(c)}(x,z) + I_z^{(b)}(y,z). \tag{3.5}$$

The functions $H_y^{(b)}(y,z)$, $I_y^{(b)}(y,z)$, $H_z^{(b)}(y,z)$, and $I_z^{(b)}(y,z)$ are calculated by Eqs. (2.35) – (2.38), while the functions $H_z^{(c)}(x,z)$ and $I_z^{(c)}(x,z)$, are calculated by Eqs. (1.51) and (1.52) at $x = R_2 + \Delta d/2$, where $\Delta d = d - R_1 - R_2$ is the distance between the surfaces of the cylinders.

It will be recalled that the amplitudes of the radial oscillation of the cylinders, A_1 and A_2, and the amplitudes of the oscillation modes of the bubbles, $s_n^{(1)}$ and $s_n^{(2)}$, are assumed to be input parameters in our theory. In other words, they should be considered as fitting parameters because their measurement is beyond our experimental capabilities. Figure 4e in our paper was calculated at $A_1 = 0$, which means that cylinder 1 does not oscillate (it only acts as a scatterer), $A_2 = 1$ μm, the amplitudes of the radial bubble modes are $s_0^{(1)} = s_0^{(2)} = 0.25$ μm, and $s_n^{(1)} = s_n^{(2)} = 0$ for $n > 0$. These parameters provide qualitative agreement with our experimental observations.

Appendix A. Mathematical formulas used in calculations

We use the following recurrence formulas for Bessel functions [9]:

$$Z_{-n}(x) = (-1)^n Z_n(x), \tag{A1}$$

$$Z_n^{/}(x)=\frac{n}{x}Z_n(x)-Z_{n+1}(x)\,, \tag{A2}$$

$$\frac{2n}{x}Z_n(x)=Z_{n-1}(x)+Z_{n+1}(x)\,, \tag{A3}$$

where Z_n stands for J_n or $H_n^{(1)}$ and the prime denotes the derivative with respect to an argument in brackets.

We also use Graf's addition theorem for Bessel functions [9],

$$Z_n(kr_j)e^{in(\theta_j-\theta_{jl})}=\sum_{m=-\infty}^{\infty}Z_{n+m}(kr_{jl})J_m(kr_l)e^{im(\pi-\theta_l+\theta_{jl})},\quad j\neq l\,, \tag{A4}$$

where Z_n can represent any of the Bessel functions, (r_j,θ_j) and (r_l,θ_l) are polar coordinates centered at two different positions, and (r_{jl},θ_{jl}) are the polar coordinates of the *l*-system with respect to the *j*-system. For $H_n^{(1)}$, Eq. (A4) is valid only provided that $r_l < r_{jl}$.

With the help of Eqs. (A1) – (A3), we obtain from Eq. (A4),

$$H_n^{(1)}(kr_1)e^{in\theta_1}=\sum_{m=-\infty}^{\infty}(-1)^{n+m}H_{n-m}^{(1)}(kd)J_m(kr_2)e^{im\theta_2}\,, \tag{A5}$$

$$H_n^{(1)}(kr_2)e^{in\theta_2}=\sum_{m=-\infty}^{\infty}H_{n-m}^{(1)}(kd)J_m(kr_1)e^{im\theta_1}\,, \tag{A6}$$

where (r_1,θ_1) and (r_2,θ_2) are polar coordinates shown in Fig. 1b and d is the distance between the centers of these coordinates.

4. The cross-section view of the interstice

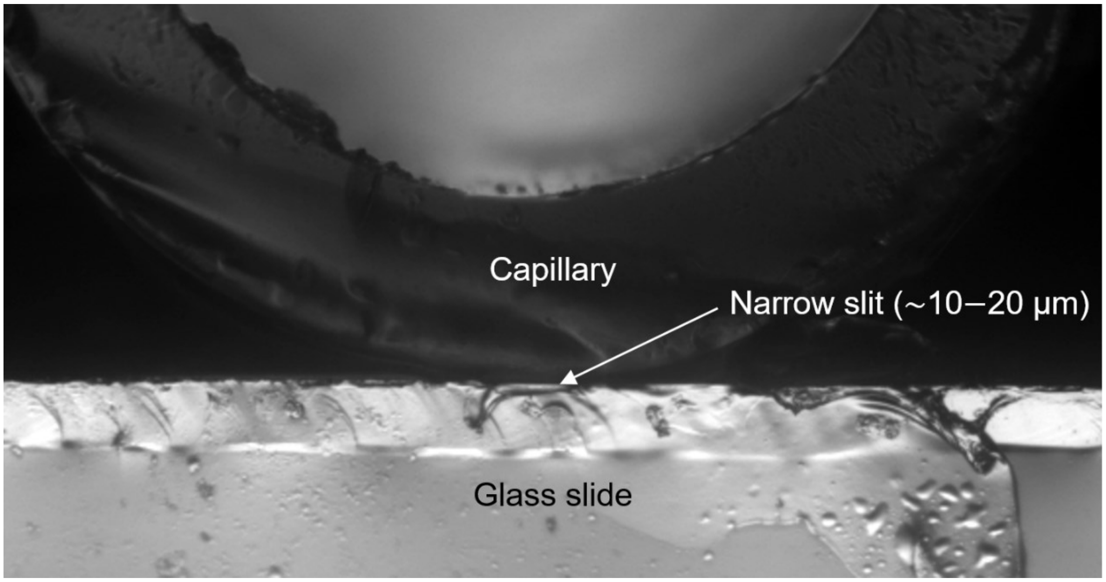

Fig. S1| The micrograph illustrates a cross-section view of the interstice, which is composed of a glass capillary pressed on top of a transparent glass slide surrounded by a shear-thinning gel.

5. Estimation of the acoustic pressure field across the slit.

Vibrometer experiments. The amplification of acoustic pressure at the slit is caused by the vibration of the glass slide (as discussed in the supplementary information). As the vibration amplitude and the pressure are directly linked by **Eq. 1.20**, we experimentally measured the vibration of the glass slide via a vibrometer. This measurement is based on the Doppler effect, with the vibrational velocity being computed by exciting a laser on the glass slide and comparing the phase shift between the incident and the reflected beams. A mapping of the vibration mode has been recorded (**Fig. S2a**) and a transfer function experimentally evaluated around the exciting frequency (**Fig. S2b**). Given the transfer function, the peak amplitude of the glass slide can be derived (~ 1 μm). Once plugged into **Eq. 1.20**, the computed theoretical pressure is around 100 kPa. Finite Element Analysis. To explore the robustness of our model and take into account the geometrical particularity of the setup (piezo transducer geometry, finite dimensions of glass slide, the capillary, and of the gel surrounding the capillary) a finite element method analysis had been performed via the software COMSOL 6.0. This analysis first corroborated the observed vibrational mode (**Fig. S3**) and for a peak amplitude displacement of 1 μm computed a pressure at the slit of ~ 30 kPa.

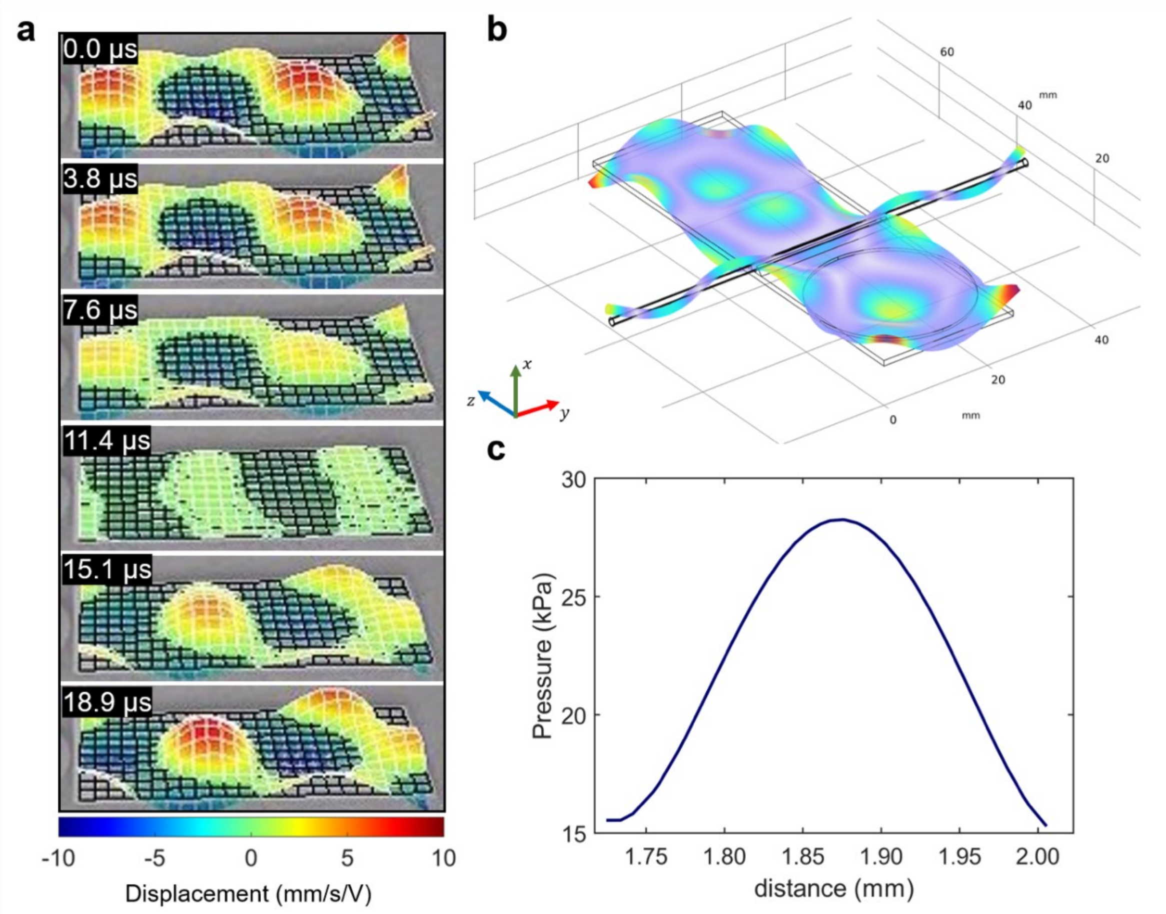

Fig. S2| a. An illustration of the vibrational mode of the glass slide over a period with an excitation frequency and amplitude of 22 kHz and 20 V_{PP}, respectively. **b.** Finite element simulation illustrates the glass slide displacement deformation at 22 kHz. **c.** A pressure versus distance plot indicates the amplification of the acoustic pressure field across the slit.

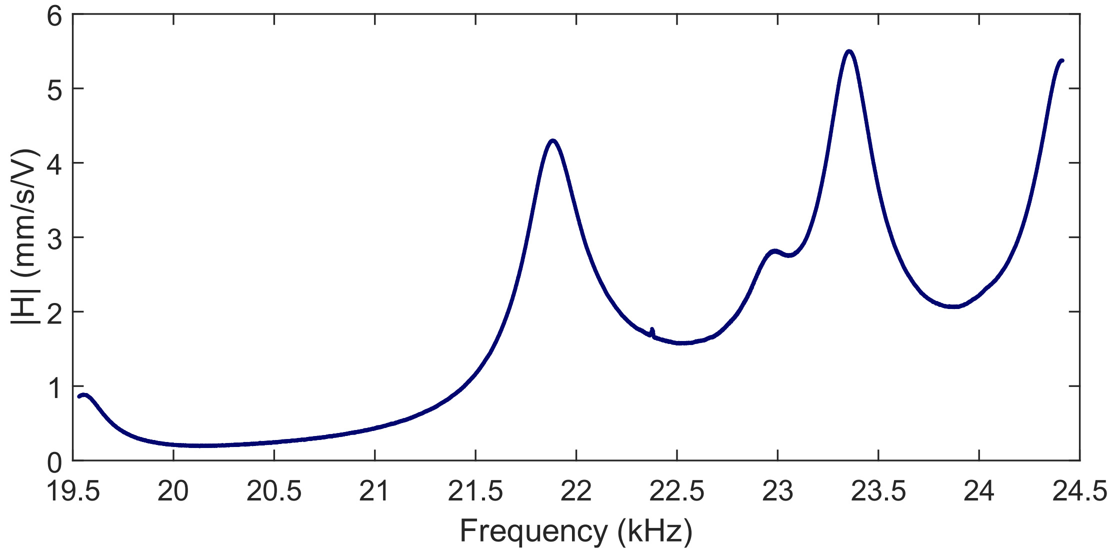

Fig. S3| Vibrometer measurement of the transfer function of a piezoelectric transducer glued to a glass slide; this function links the excitation frequency to glass slide velocity displacement per volt applied.

6. Nucleation and self-assembly of microbubbles.

Initially, the slit-liquid interface at the edge is where cavitation bubbles are most readily formed due to the simultaneous high-density distribution of gas nuclei and high acoustic pressure; this can be considered as the place of origin of the bubbles, **Fig. S4**. As these bubbles of a few microns in diameter continue to be created, adjacent bubbles are attracted to each other and merge into one large bubble. These larger bubbles are then gathered by acoustic pressure to the centreline and stabilized into a bubble train.

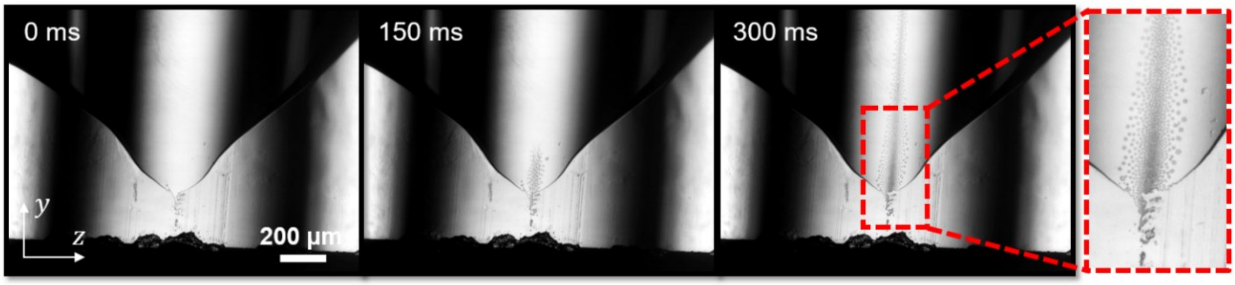

Fig. S4| The generation and evolution of a microbubble train. a. Stage 1: Cavitation starts at the edge of the gel (gas-liquid interface), forming microbubbles that move toward the centre. b. Stage 2: Adjacent microbubbles gather and merge continuously into one large bubble. c. Stage 3: The large bubbles gather towards the centreline position and finally stabilize into a bubble train.

7. Nucleation of microbubbles in KY Gel, Polydimethylsiloxane (PDMS) solution, and Epoxy Resin glue.

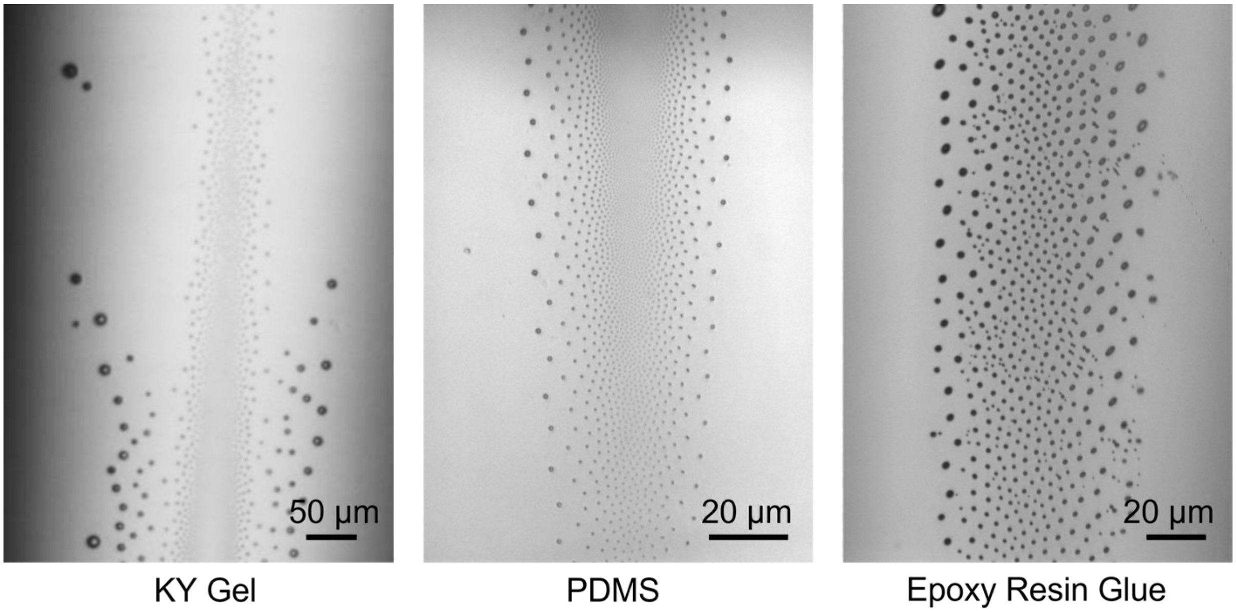

Fig. S5| Nucleation of microbubbles in KY Gel, Polydimethylsiloxane (PDMS) solution, and Epoxy Resin glue when acoustically stimulated at ~23 kHz and 20 V_{PP}.

Materials	KY Gel	PDMS	Epoxy Resin Glue
Viscosity	0.1-100 Pa·s	1.8-2.6 Pa·s	10-15 Pa·s
Density	1 g/cm³	0.97 g/cm³	1.17g/cm³

Tab. S1| The viscosity and density of KY Gel, PDMS, and Epoxy resin glue.

8. The size and shape of microbubbles versus driving power

As the microbubble stabilizes along the centerline, increasing the drive voltage alters the microbubble's shape (morphology). **Fig. S6** shows the aspect ratio variation of the bubble with an increase in voltage. The microbubble stretches along the centerline as the voltage increases, i.e., from 25– 40 V_{PP}. However, when the acoustic pressure or voltage exceeds 40 V_{PP}, the bubble behaves erratically, i.e., the microbubble starts to distort, and satellite microbubbles are nucleated at higher voltage.

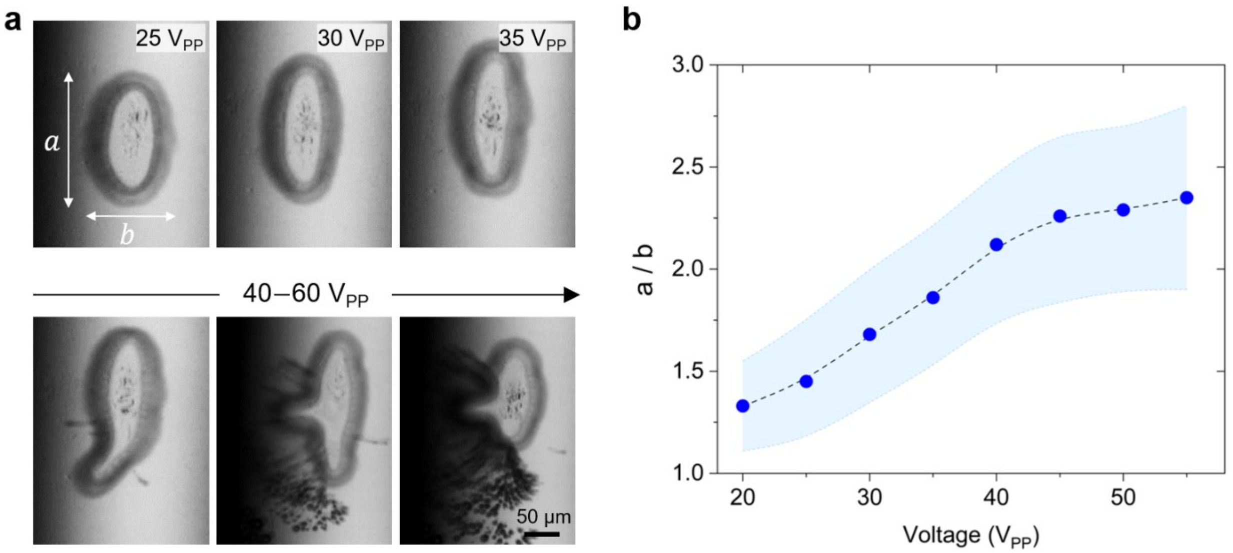

Fig. S6| a. Image sequence demonstrate the morphology of the microbubble versus driving voltage. **b**. A plot shows the microbubble's size as a function of driving voltage. Each data point represents the average distance measured from at least three locations. Error bars in both graphs b represent standard deviation (s. d.) as $n \leqslant 4$. See Supplementary Data Files 1 for the source data for the graph.

9. Propulsion along a narrow slit at different capillary orientations

We have performed additional experiments by rotating the capillary from 0 to 60 degrees, as shown in **Fig. S7**. We observed the microbubbles to self-assemble into a train-like arrangement and propel in both directions regardless of the angle at which the capillary was positioned. We can therefore assume that the direction of travel along the capillary rail is independent of capillary position. Furthermore, we have flipped the setup to investigate the effect of gravity, but no significant changes were observed. The microbubbles consistently self-assemble and propel when acoustically activated.

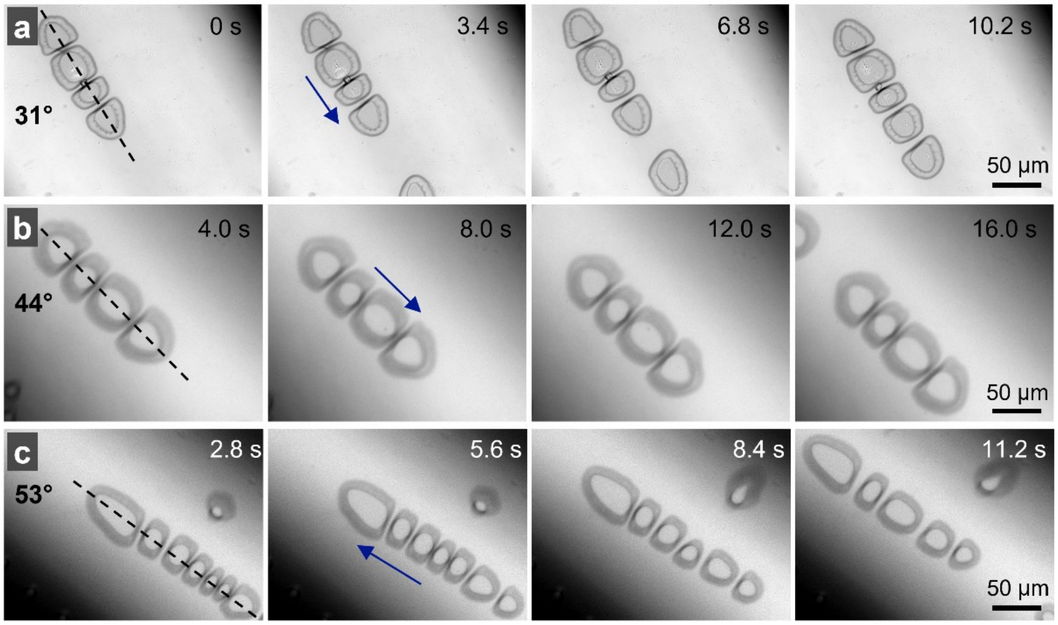

Fig. S7| Image series demonstrating bubble train propulsion along a narrow slit at three different capillary orientations when acoustically activated at 22.3 KHz and 20 V_{PP}: **a.** 31^0, **b.** 44^0, and **c.** 53^0.

10. The microbubble train over long distances

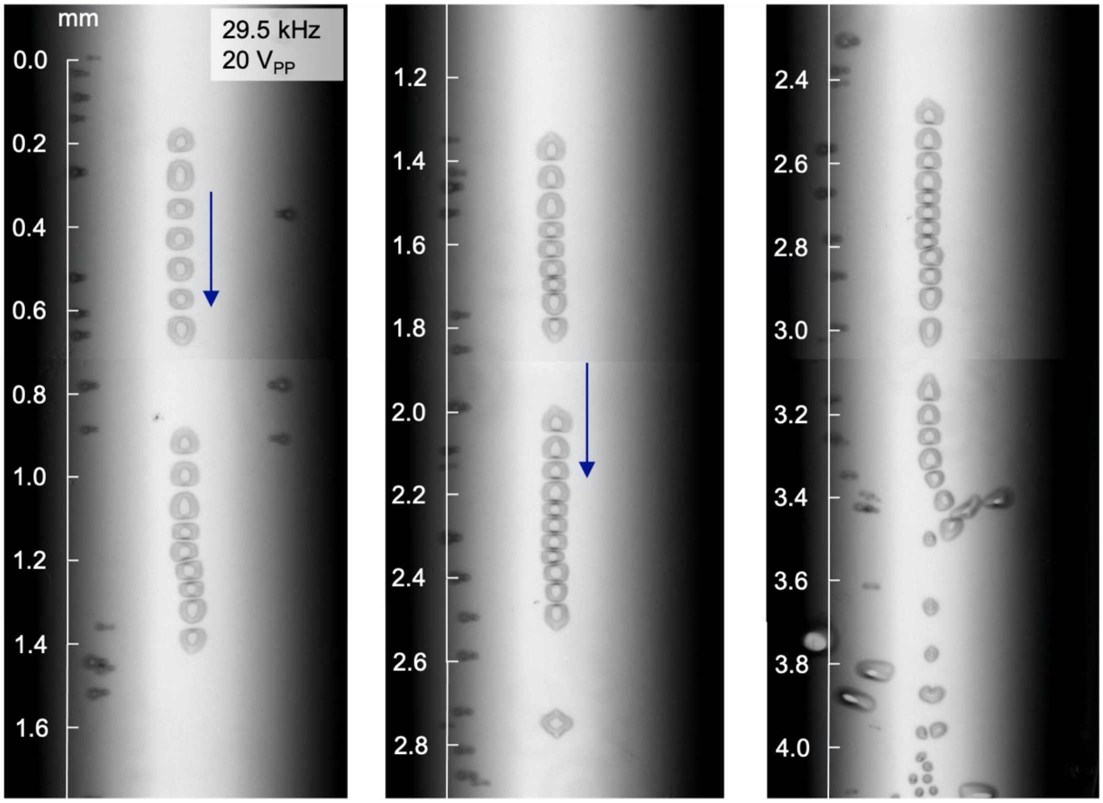

Fig. S8| Image sequences demonstrate propulsion of the microbubble train over long distances (~3.5 mm) when acoustically activated at 29.5 kHz and 20 V_{PP} (see also **Supplementary Video 8**). When the microbubble train reaches the edge of the glass slide, it begins to disassemble.

11. An investigation of the number of bubbles self-assembling in a linear array

The number of microbubbles in a train is studied as a function of excitation voltage. **Fig. S9** demonstrates that the number of microbubbles in a train varies between five and six as the acoustic voltage increases from 20 to 40 V_{PP} at 22.6 kHz. However, no direct correlation has been observed for increasing voltage. The number of microbubbles in a train versus excitation frequency is investigated. Microbubbles and microbubble trains are produced at certain excitation frequency bands, which coincide with the piezo transducer's resonance frequencies. Figure illustrates that the number of microbubbles in a train remains between five and six. The experiments confirmed that the input voltage and the different excitation frequencies do not significantly influence the number of bubbles in the bubble train.

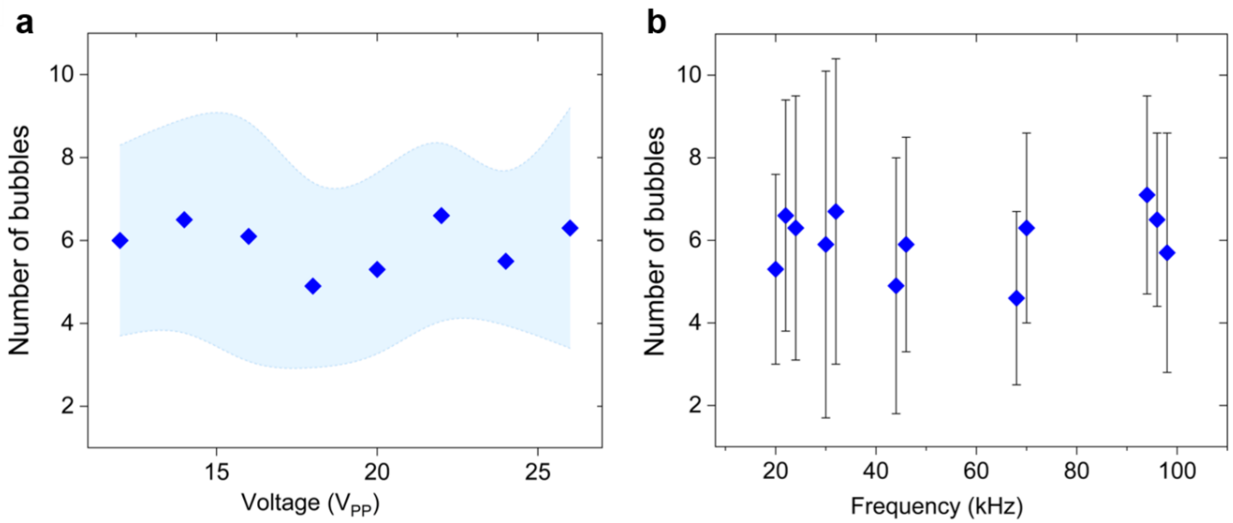

Fig. S9| The number of microbubbles in a train as a function of driving frequency and power. Each data in both graphs **a.** and **b.** represents the average number of microbubbles assembled in a linear array analyzed from 3-5 train-like assembly (Source Data). Error bars represent the standard deviation (s. d.) of data. See Supplementary Data File 1 for the source data for the graph.

12. Interparticle distance of adjacent microbubbles as a function of acoustic voltage.

The interparticle distance, *d*, between adjacent microbubbles in a train-like assembly for a sweeping voltage of 16.8 to 18 V_{PP} at a fixed excitation frequency of 23.2 kHz is characterized. **Fig. S10** illustrates with increasing piezotransducer voltage, the interparticle distance between adjacent microbubbles in the train decreases. As the oval-shaped microbubble gets closer to adjacent microbubbles, it transforms into a rectangular shape. And a further increase in the applied power ejects a neighboring microbubble.

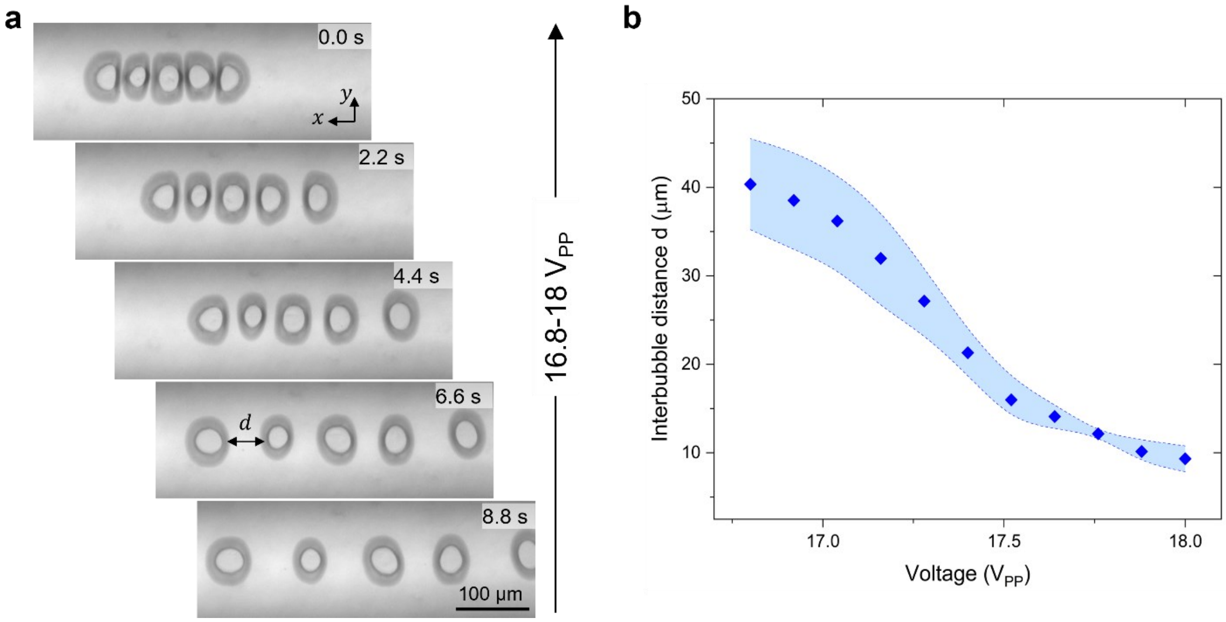

Fig. S10| a. Characterization of the interparticle distance, d, between adjacent microbubbles in a train-like assembly for a sweeping voltage of 16.8 to 18 V_{PP} at a fixed excitation frequency of 23.2 kHz. **b.** The plot shows the interparticle distance with respect to the driving voltage**.** Each data point represents the average distance measured from at least three different locations. Error bars in both graphs **b**. represent standard deviation (s. d.) as $n \leqslant 4$. See Supplementary Data Files 1 for the source data for the graph.

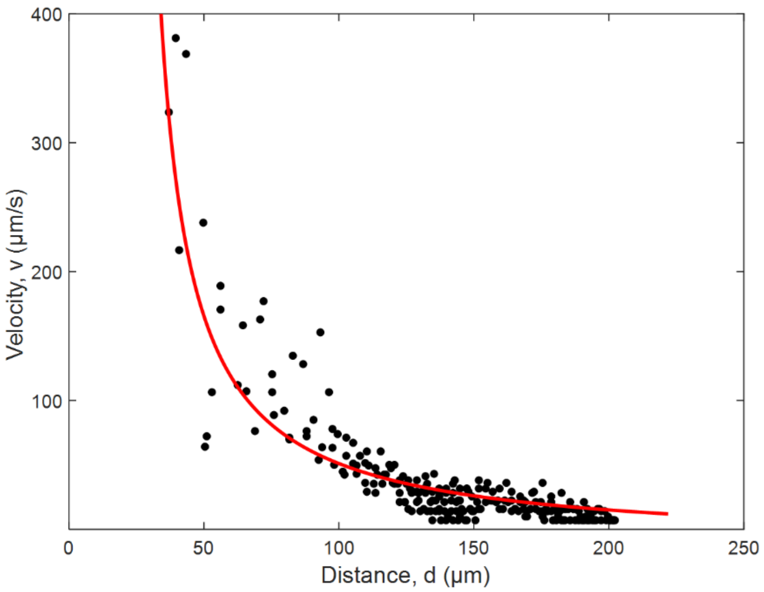

Fig. S11| Analysis of a microparticle trap within ellipsoidal microbubbles. Plot showing the velocity of microparticles as they migrate towards an oscillating ellipsoidal microbubble. The red colour indicates the fit.

13. Trapping of magnetic microparticles in the microbubble train.

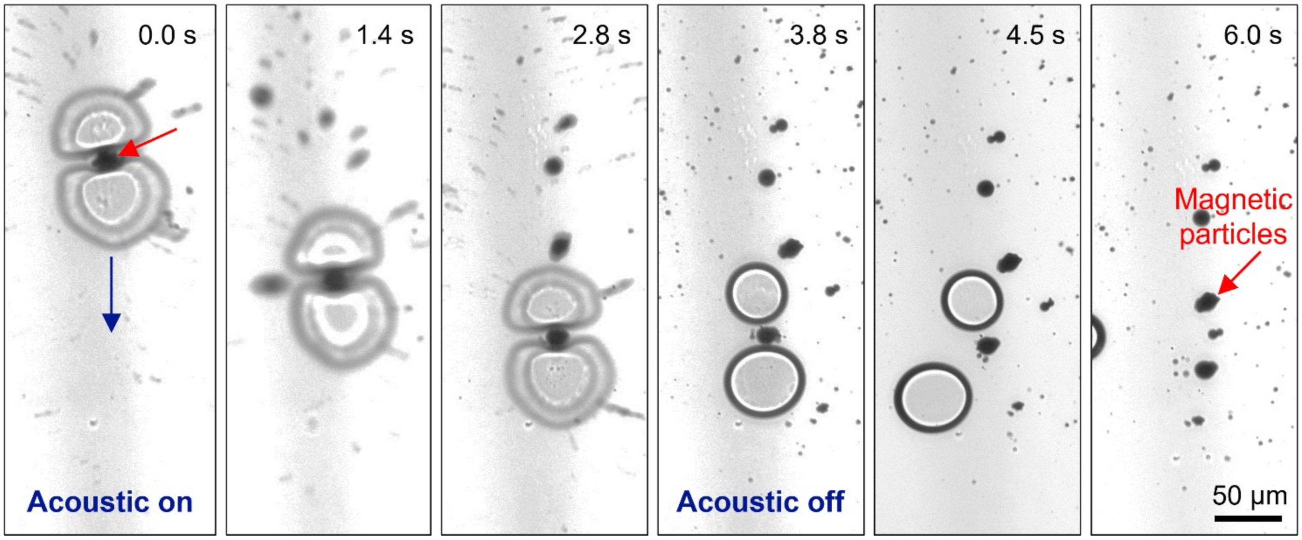

Fig. S12| Image sequences demonstrate the trapping, transport, and release of superparamagnetic microparticles in gels when acoustically activated at ~23 kHz and 20 V_{PP}.

14. The rheological properties of shear-thinning viscous gel.

The rheological properties of shear-thinning gels were investigated experimentally.

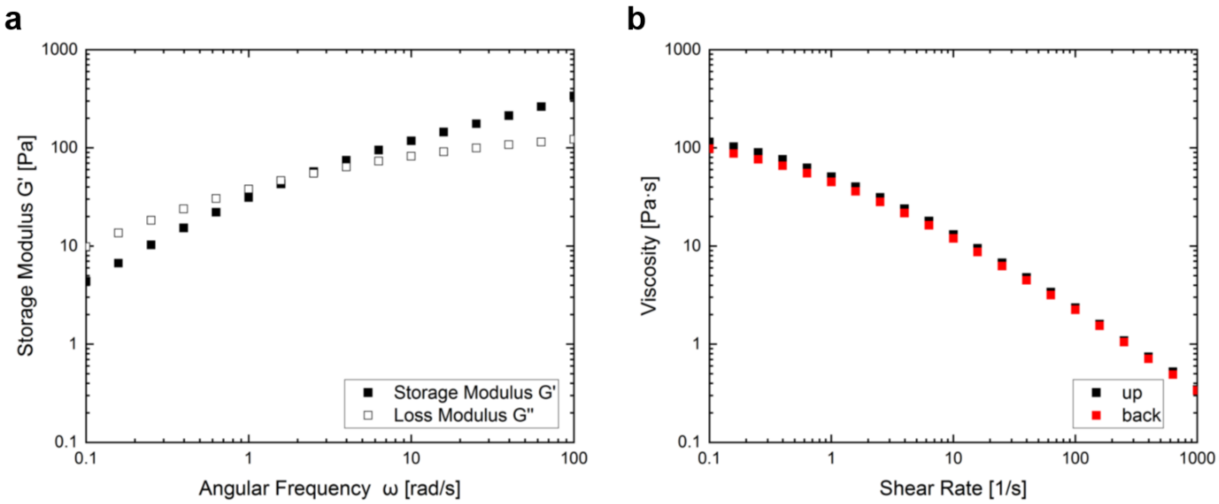

Fig. S13| Experimentally measured flow curves for KY gel **a.** Storage modulus versus angular frequency; **b.** Viscosity versus shear rate.

References

[1] L. D. Landau and E. M. Lifshitz, *Fluid Mechanics* (Pergamon Press, Oxford, 1987).
[2] P. M. Morse and H. Feshbach, *Methods of Theoretical Physics* (McGraw-Hill, New York, 1953), Part I.
[3] T. G. Leighton, The Acoustic Bubble (Academic Press, San Diego, 1994).
[4] L. Rayleigh, On the pressure developed in a liquid during the collapse of a spherical cavity, Phil. Mag. **34**, 94 (1917).
[5] M. S. Plesset, The dynamics of cavitation bubbles, J. Appl. Mech. **16**, 277 (1949).
[6] L. P. Gor'kov, On the forces acting on a small particle in an acoustical field in an ideal fluid, Sov. Phys. Dokl. **6**, 773 (1962).
[7] D. A. Varshalovich, A. N. Moskalev, and V. K. Khersonskii, *Quantum Theory of Angular Momentum* (World Scientific, Singapore, 1988).
[8] A. A. Doinikov and A. Bouakaz, Theoretical model for coupled radial and translational motion of two bubbles at arbitrary separation distances, Phys. Rev. E **92**, 043001 (2015).
[9] M. Abramowitz and I. A. Stegun, *Handbook of Mathematical Functions with Formulas, Graphs and Mathematical Tables* (Dover, New York, 1972).
